# Distinct transcriptomic and epigenomic modalities underpin human memory T cell subsets and their activation potential

**DOI:** 10.1038/s42003-023-04747-9

**Published:** 2023-04-03

**Authors:** James R. Rose, Bagdeser Akdogan-Ozdilek, Andrew R. Rahmberg, Michael D. Powell, Sakeenah L. Hicks, Christopher D. Scharer, Jeremy M. Boss

**Affiliations:** 1 Department of Microbiology and Immunology, and the Emory Vaccine Center, Emory University School of Medicine, Atlanta, GA 30322 USA; 2 Barrier Immunity Section, Laboratory of Viral Diseases, Division of Intramural Research, National Institute of Allergy and Infectious Diseases, NIH, Bethesda, MD USA

**Keywords:** Epigenetics in immune cells, Molecular biology

## Abstract

Human memory T cells (MTC) are poised to rapidly respond to antigen re-exposure. Here, we derived the transcriptional and epigenetic programs of resting and ex vivo activated, circulating CD4^+^ and CD8^+^ MTC subsets. A progressive gradient of gene expression from naïve to T_CM_ to T_EM_ is observed, which is accompanied by corresponding changes in chromatin accessibility. Transcriptional changes suggest adaptations of metabolism that are reflected in altered metabolic capacity. Other differences involve regulatory modalities comprised of discrete accessible chromatin patterns, transcription factor binding motif enrichment, and evidence of epigenetic priming. Basic-helix-loop-helix factor motifs for AHR and HIF1A distinguish subsets and predict transcription networks to sense environmental changes. Following stimulation, primed accessible chromatin correlate with an augmentation of MTC gene expression as well as effector transcription factor gene expression. These results identify coordinated epigenetic remodeling, metabolic, and transcriptional changes that enable MTC subsets to ultimately respond to antigen re-encounters more efficiently.

## Abstract (web summary)

An integrated analysis uncovers transcriptional and epigenetic differences of human circulating memory T cell subsets and identifies unique transcription factor networks associated with memory subset differentiation.

## Introduction

Memory T cells (MTC) arise after a naïve T cell responds to an initial interaction with antigen and play an essential role in mounting a robust secondary response upon reinfection. These cells, composed of both CD4 and CD8 expressing T cells, exhibit enhanced effector functions and a heightened response to subsequent activations with antigen^1^. Human MTC are extremely long-lived, with one study finding antigen-specific T cells still detectable in individuals 75 years after vaccination, suggesting a capacity for self-renewal not seen in shorter-lived effector cells^2^. Naïve and memory CD4^+^ and CD8^+^ T-cell subsets are commonly subdivided based on cell surface expression of CCR7, CD45RA, and CD62L into naïve (Nav, CCR7^+^CD45RA^+^), central memory (T_CM_, CCR7^+^CD45RA^–^), effector memory (T_EM_, CCR7^–^CD45RA^–^), and terminally differentiated effector memory (T_EMRA_, CCR7^–^CD45RA^+^)^3,4^. CCR7 and CD62L are highly expressed in T_CM_ and naïve T cells and enable cellular homing to secondary lymphoid organs. T_EM_ exhibit lower expression of both CCR7 and CD62L and are thought to instead localize to inflamed tissues where they exhibit a higher degree of effector function^5^. Additionally, naïve T cells can be separated from T_EM_ and T_CM_ cells by higher expression of the CD45RA isoform^6^, which is downregulated in T_CM_ and T_EM_ cells. However, a subset of effector-memory-like human CD8^+^ T cells in the blood express high levels of CD45RA (CCR7^–^CD45RA^+^) while simultaneously expressing effector function genes such as *GZMB* and *PRF1* (encoding granzyme B and perforin, respectively)^7^. These cells (T_EMRA_) are believed to be highly differentiated effector cells or potentially even senescent versions of effector MTC, which arise after chronic infections with virus^8–10^. Despite their importance in immunological memory, the full spectrum of transcriptional and epigenetic characteristics of these main memory subsets, the transcription factor programs associated with each, and how they play a role in memory responses remains to be fully understood.

Previous studies have profiled various epigenetic and transcriptional aspects of CD8^+^ MTC^11–13^; however, the full phenotypic and epigenetic characteristics of the subsets found within the entire circulating human MTC compartment remains largely under-defined. At the same time, changes to the transcriptomic and epigenetic landscape of T cells after initial activation of naïve cells have been shown to be important in both establishing the differentiated memory subsets, and in rapid recall response upon re-stimulation^14–16^. Understanding how these memory-subset-specific epigenetic changes affect their formation and function upon re-encounter with antigen will ultimately inform more effective therapeutic design.

To better understand the epigenomic parameters of human MTC subsets and how such parameters dictate gene expression, we investigated transcriptional and epigenetic differences between CD4^+^ and CD8^+^ memory cell subtypes (T_CM_, T_EM_, and T_EMRA_) from human blood, as well as in response to ex vivo stimulation. Analysis of changes in mRNA transcripts and chromatin accessibility revealed that MTC share a substantial set of genes expressed at similar levels irrespective of linage and cell subset. Moreover, we observed evidence of a progressive increase in the amount of differentiation from naïve T cells, to T_CM_, and finally to T_EM_ and T_EMRA_ memory populations. One set of genes that were upregulated, included those that reflected changes in metabolic capacity of the MTC subsets. Biochemical analyses confirmed differences in the metabolic capacity of various MTC subsets. Combining gene expression and chromatin accessibility analyses before and after stimulation identified a series of patterned regulatory modalities that may define memory-subset differentiation, as well as increased reactivity in response to secondary activations. Such dynamic regions of chromatin were enriched for motifs known to bind a small group of transcription factors (TFs) from the basic leucine zipper (bZIP), high mobility group (HMG), T-box, and basic helix-loop-helix (bHLH) families. Altogether, these data lay out the full spectrum of transcriptional and epigenetic differences of the primary memory-subset categories found in human blood, while also identifying the unique transcription factor networks associated with memory-subset differentiation and highlighting loci that may be important in establishing a memory cell’s response to activation upon secondary antigen exposure.

## Results

### The shared transcriptional programs of human CD4^+^and CD8^+^ MTC

**Fig. 1:**
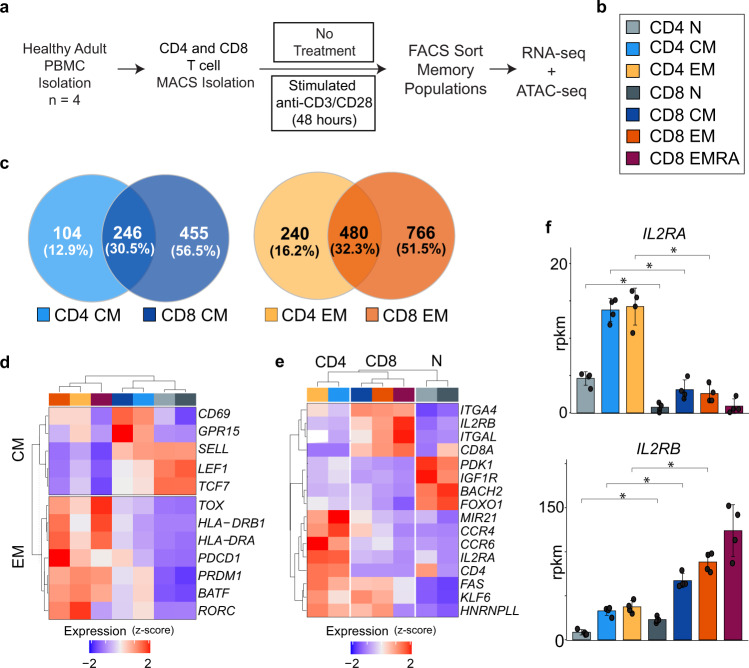
MTC display distinct patterns of shared and differentially expressed genes that define their subsets and lineage. **a** Experimental workflow for isolation, ex vivo stimulation, and sequencing analysis of MTC from human blood samples. **b** Color code of naïve T cell and MTC lineages and subsets used throughout are shown. **c** Venn diagrams representing total number of DEG for T_CM_ and T_EM_ compared to naïve T cells. **d** Heatmap and hierarchal clustering of gene expression for select genes commonly expressed in either T_CM_ or T_EM_ regardless of lineage. **e** Heatmap of lineage-specific gene expression in MTC. **f** Gene expression bar plots (reads per kilobase million, rpkm) for indicated IL-2 receptor genes. Data are plotted as mean ± SD (standard deviation); asterisks indicate DEG (FDR ≤ 0.05) as detected by DESeq2 algorithm. Cell type color codes are shown in (**b**).

To define the transcriptional profiles of the major subsets of MTC, naïve, T_CM_, and T_EM_ cells from CD8^+^ and CD4^+^ lineages were FACS separated from the blood of four human donors using CCR7 and CD45RA as distinguishing cell surface markers (Fig. 1a, b**, and** Supplemental Fig. 1a). Naïve T cells were defined as CCR7^+^CD45RA^+^; T_CM_ as CCR7^+^CD45RA^–^; and T_EM_ as CCR7^–^ CD45RA^–^. CD8^+^ T_EMRA_ T cells (CD45RA^+^CCR7^–^), a terminal effector subset was also included for comparison (Supplemental Fig. 1b-c). To elucidate transcriptional differences between naïve and memory subsets RNA-sequencing (RNA-seq) was conducted on sorted populations. The mRNA content of each sorted subset group was assessed for shared and unique transcripts defining MTC from naïve T cells. Relative to their naïve counterparts, T_CM_ and T_EM_ cells exhibited 805 and 1,486 differentially expressed genes (DEG), respectively (Fig. 1c, Supplemental Data 1). Comparing subsets between lineages (CD4^+^ vs. CD8^+^) showed that T_EM_ and T_CM_ cells each shared approximately 30% of their DEG, highlighting conserved relationships between the CD4^+^ and CD8^+^ lineages. Gene ontology analysis of the lineage specific and shared DEG showed that genes shared between lineages were highly enriched for pathways related to T-cell activation, co-stimulation, lymphocyte homeostasis, and cytokine response/production in both T_CM_ (Supplemental Fig. 2a) and T_EM_ subsets (Supplemental Fig. 2b). T_EM_ DEG shared between CD4^+^ and CD8^+^ T cells were also more highly enriched for antigen processing and presentation, as well as GTPase signaling pathways.

Examples of genes expressed in both lineages included the transcription factors *LEF1* and *TCF7*, which were more highly expressed in both CD4^+^ and CD8^+^ T_CM_ and naïve T cells. *PDCD1*, encoding PD-1, and MHC class II genes encoding HLA-DR were expressed higher in T_EM_ cells of both lineages (Fig. 1d). Other genes exhibited lineage-specific expression patterns, including integrins (e.g., *ITGAL*/CD11a, *ITGA4*/VLA4), which were expressed more highly in CD8^+^ MTC; whereas the chemokine receptors *CCR4* and *CCR6*, which have been implicated in homing to specific peripheral tissues such as the skin^17,18^, were more expressed in CD4^+^ MTC (Fig. 1e). Lineage-specific DEG encoding components of the interleukin-2 receptor (IL2R)^19^ were also observed, with CD4^+^ MTC expressing higher levels of the α chain (*IL2RA*, Fig. 1f), while CD8^+^ MTC expressed considerably higher levels of *IL2RB* (Fig. 1f). These differences emphasize the fact that while all MTC share important gene pathways, which make them unique from naïve cells, transcriptional differences can also distinguish each lineage and/or subset underlying their distinct immunological functions.

### Resting T_EM_/T_EMRA_ cells exhibit progressively greater transcriptional differentiation from naïve progenitors than T_CM_

**Fig. 2:**
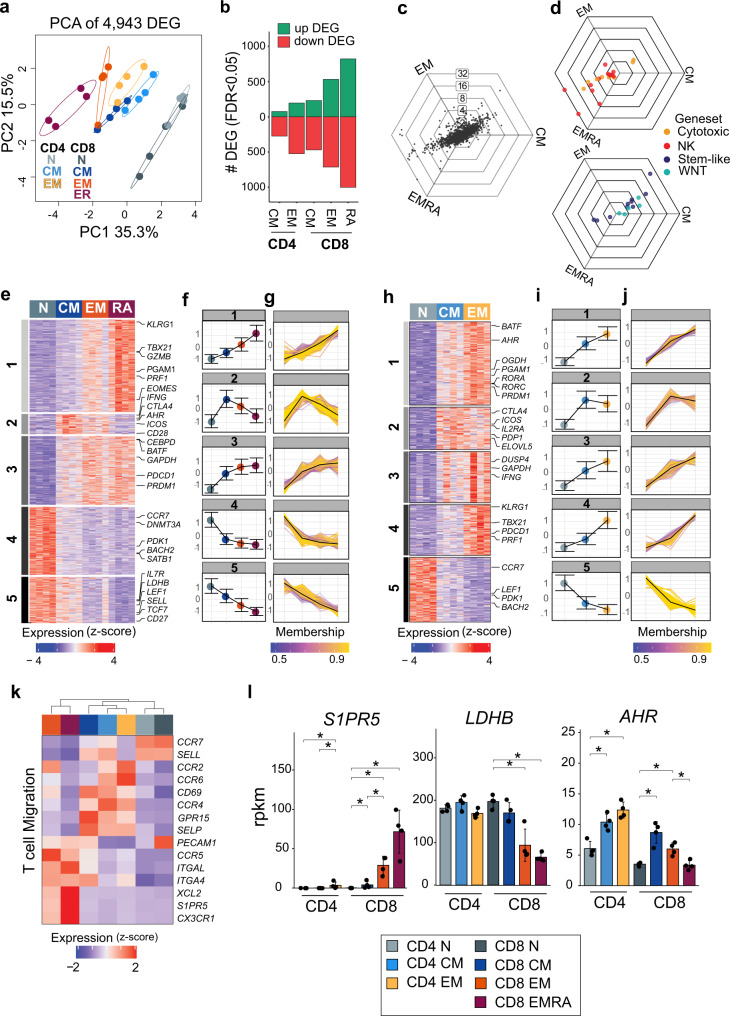
Resting MTC subsets in peripheral blood show progressive levels of differentiation from naïve. **a** Principal component (PC) analysis plot of DEG from RNA-sequencing data showing each sample separated by first two components. **b** Bar plot representing number of up and downregulated DEG for each memory subset compared to naïve T cells. **c** Three-way representation of DEG expression between CD8^+^ T_CM_, T_EM_, and T_EMRA_. Each dot represents a single DEG; dots falling directly on an axis represent exclusive upregulation in each group, and concentric rings represent degree of log_2_-fold-change differences between groups. **d** Overlay of C with genes belonging to two gene sets (NK cell and cytotoxicity) upregulated in CD8^+^ T_EM_ and T_EMRA_ cells, or genes representing WNT-Beta catenin and stem-like T-cell expression are upregulated in CD8^+^ T_CM_ cells. **e,** **h** Heatmaps showing DEGs in CD8 + or CD4 + naïve and memory subsets clustered by fuzzy c-means clustering. Highlighted examples for each module are listed. **f,** **i** Line plots showing mean expression of all genes in each cluster by subset (error bars represent ±1 SD). **g,** **j** Line plots showing expression of individual genes in cluster colored by membership score for respective cluster. Average across modules shown by black line. **k** Heatmap of selected genes representing differences in expression of migration-related genes. Data represents the mean expression of each cell subset. **l** Gene expression bar plots showing reads per kilobase million (rpkm) for the indicated genes. Data are plotted as mean ± SD; asterisks indicate DEG as detected by DESeq2 algorithm.

A total of 4,943 DEG distinguished naïve cells from CD4^+^ and CD8^+^ MTC subsets. Principal component analysis (PCA) of these DEG indicated that the bulk of this variation separated naïve T cells from T_CM_, T_EM_, and T_EMRA_ subsets (Fig. 2a). Differentiation from naïve cells showed progressive increases in both up and down DEG from T_CM_ to T_EM_ in both CD4^+^ and CD8^+^ T cells and finally to T_EMRA_ in CD8^+^ T cells subsets (Fig. 2b). Fold-change levels of DEG in CD8^+^ T-cell memory subsets were compared using hexagonal “tri-wise” visualization (Fig. 2c)^20^. These plots contain three axes corresponding to each subset and each radiating axis represents fold-change levels. DEG that fall directly on each axis represent those expressed exclusively by that subset, while those sharing high fold-change levels across two groups relative to the third subset fall midway between spokes. This analysis revealed that many of the DEG with highest fold change were shared between T_EM_ and T_EMRA_ subsets but absent in T_CM_ cells. Overlaying genes from several gene ontology gene sets relevant to MTC showed that genes involved in pathways related to cell cytotoxicity as well as NK-related genes involved in cell killing were exclusively upregulated in T_EM_ and T_EMRA_ subsets (Fig. 2d). Alternatively, a custom gene set representing genes expressed by stem-like T cells derived from Hudson et al. ^21^, as well as gene ontology gene sets representing the WNT-beta catenin pathway were highly upregulated in the T_CM_ subset relative to either effector subset (Fig. 2d).

Fuzzy c-means clustering of the DEG was used to identify gene modules across the CD8^+^ and CD4^+^ MTC subsets. Fuzzy c-means clustering allows genes to be assigned membership to multiple overlapping clusters before an ultimate single cluster classification is determined by ranking the magnitude of membership score for that gene. This analysis identified five distinct modules of gene expression programs within the DEG of CD8^+^ T cells across all memory subsets (Fig. 2e, Supplemental Data 2). Modules 1 and 3 corresponded to genes that were upregulated in CD8^+^ T_EM_ and T_EMRA_ cells compared to naïve T cells. Gene ontology analysis showed that genes in module 1 were most highly expressed in T_EMRA_ cells and enriched for pathways related to cell killing, cytolysis, and innate immune response (Supplemental Fig. 3a). Module 3 genes were expressed equally in CD8^+^ T_EM_ and T_EMRA_ cells and enriched for pathways related to apoptosis, MAPK activity, and tyrosine kinases (Supplemental Fig. 3b). Modules 4 and 5 represented genes repressed in CD8 + T_EM_ and T_EMRA_ subsets but more highly expressed in naïve or T_CM_ cells (Fig. 2f) with individual genes plotted and colored according to cluster membership (Fig. 2g). These were enriched for DNA methyltransferases such as DNMT3A or pathways related to T-cell differentiation, respectively (Supplemental Figs. 3c, d). Module 2 contained fewer genes than any of the other modules and included those expressed in both CD8^+^ T_CM_ and T_EM_ memory subsets with low expression in both naïve and T_EMRA_ cells. This module was highly enriched for pathways related to T-cell receptor activation, co-stimulation, and IL-2 production (Supplemental Fig. 3e).

Applying the same clustering method to CD4^+^ subsets showed a similar pattern of clustering with five total gene modules (Fig. 2h, i, Supplemental Data 3) with individual genes plotted (Fig. 2j). Modules 1, 3, and 4 were most highly expressed in CD4^+^ T_EM_ cells (Fig. 2i) but showed differences in gene ontology pathway enrichment (Supplemental Fig. 3f-h). These included secretory granules and carbohydrate metabolism (module 1), cytokine secretion, and T-cell activation (module 3), and MAP Kinase activity or cytolysis (module 4). The expression of genes in module 5 was highest in CD4^+^ naïve T cells and contained similar gene set enrichments to module 5 in CD8^+^ MTC (Supplemental Fig. 3i), including self-renewal related transcription factors, such as *LEF1*. Module 2 was expressed in both CD4^+^ T_CM_ and T_EM_ subsets as in the CD8^+^ lineage. Pathway enrichment for this module showed enrichment for several metabolism-related pathways including cofactor and lipid biosynthesis (Supplemental Fig. 3j). As with CD8^+^ MTC, important co-stimulatory/co-inhibitory molecules, such as CTLA4 and ICOS, were also present in CD4^+^ module 2 and expressed by both T_CM_ and T_EM_ subsets. Thus, both CD4^+^ and CD8^+^ lineages showed a progression of increasing gene expression changes relative to naïve in T_CM_, T_EM_, and finally T_EMRA_ MTC subsets.

### MTC subsets exhibit distinct migration and metabolism characteristics

**Fig. 3:**
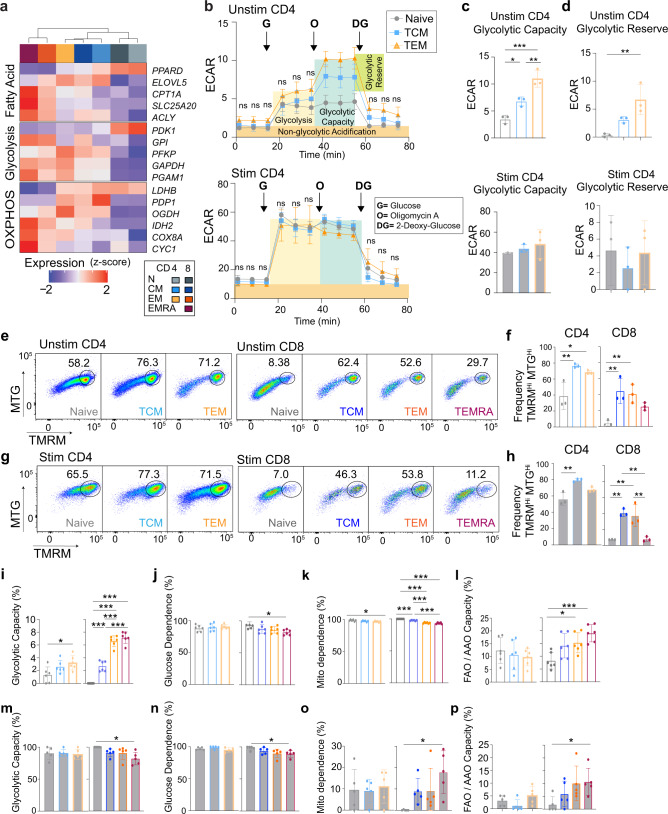
MTC subsets exhibit distinct metabolic features. **a** Heatmap of selected genes representing differences in metabolism. Data represent the mean expression of each cell subset. **b** Line plot showing Seahorse-derived ECAR data (mpH / min / 10^5^ cells) over time for unstimulated and stimulated naïve, T_CM_, and T_EM_ CD4^+^ T cells. Shaded regions indicate regions of metabolic relevance as labeled. Arrows indicate time of inhibitor addition (G, glucose; O, oligomycin; and DG: 2-deoxy-glucose). **c,** **d** Bar plots showing glycolytic capacity and glycolytic reserve calculated for unstimulated and stimulated CD4^+^ T cells, respectively. Error bars indicate ±SD. **e** Representative flow cytometry plots showing gating strategy for unstimulated cells using MitoTracker green (MTG) and tetramethylrhodamine methyl (TMRM). **f** Bar plots showing frequency of MTG^HI^TMRM^HI^ cells in naïve and MTC subsets. **g** Flow cytometry plots as in (**e**) for stimulated cells. **h** Bar plots showing frequencies as in (**f**). Bar plots showing metabolic attributes calculated from SCENITH data as indicated for unstimulated (**i**–**l**) and stimulated (**m**–**p**) cell types. Error bars indicate ±SD. For all experiments, 3–6 independent samples were analyzed. One-way ANOVA, with multiple comparisons were used to determine significance. *P < 0.05, **P < 0.01, ***P < 0.001.

Contained within the above modules were genes representing the potential for important functional differences across MTC subsets, including genes involved in T-cell migration and metabolism. For example as expected^3^, CD4^+^ and CD8^+^ naïve and T_CM_ cells expressed high levels of both *CCR7* and *SELL* (L-selectin). Whereas CD4^+^ T_CM_ and T_EM_ exclusively expressed *CCR4* and *CCR2* (Fig. 2k). CD8^+^ T_EM_/T_EMRA_ cells had the highest expression of *S1PR5*, which has been shown to be associated with promoting egress of lymphocytes from secondary lymphoid organs or bone marrow (Fig. 2l)^22^. MTC in general were found to have upregulated a greater number of metabolism-related genes compared to resting naïve T cells (Fig. 3a). Subset-specific differences were observed in expression of genes responsible for fatty acid metabolism, glycolysis, and oxidative phosphorylation (Fig. 3a). Many of these genes relate to the regulation of acetyl CoA or lactate metabolism as exemplified by expression differences of the genes *PDK1*, *PDP1*, and *LDHB*. For example, in CD8^+^ T_EM_ and T_EMRA_ cells (Fig. 2j), *LDHB*, which encodes lactate dehydrogenase enzyme subunit B has been previously associated with aerobic glycolytic metabolism in effector T cells, as well as in cancer^23,24^.

Several assays to measure and compare the metabolic states of naïve and MTC were performed. Using the Seahorse-based Glycolysis Stress Test assay on unstimulated CD4^+^ T-cell subsets, an extracellular acidification rate (ECAR) was determined (Fig. 3b). In response to glucose, CD4^+^ MTC did not exhibit a significant difference in glycolysis; however, T_CM_ and T_EM_ populations showed significantly higher glycolytic capacity (Fig. 3c) and glycolytic reserve (Fig. 3d) compared to naïve CD4^+^ T cells, suggesting that they have higher potential to increase ATP production via glycolysis under stress or other physiologically energy-demanding conditions. Anti-CD3/CD28 bead stimulated CD4^+^ T-cell subsets showed an elevated level of glycolysis compared to unstimulated cells, which was consistent with metabolic reprogramming phenomena in response to stimulation (Fig. 3b). No significant difference between the stimulated MTC subsets regarding glycolysis, glycolytic capacity, or glycolytic reserve was observed, suggesting that regardless of subset, CD4^+^ MTCs can achieve similar glycolytic metabolic rates.

Due to the limitations in obtaining purified subset cell numbers, we could not perform a similar set of assays on CD8^+^ T cells. Instead, two flow cytometry-based assays were used to assess oxidative phosphorylation (OXPHOS) and fatty acid metabolic states of both the CD4^+^ and CD8^+^ T cells (Fig. 3e–h). In the first assay, cells were stained with MitoTracker Green (MTG), a mitochondrial specific dye that provides a relative assessment of mitochondrial mass, and tetramethylrhodamine methyl (TMRM), an OXPHOS marker that accumulates in functional mitochondria caused by differential membrane potential^25,26^. Unstimulated T_CM_ and T_EM_ populations had higher frequencies of cells with highly functional mitochondria (MTG^Hi^ and TMRM^Hi^) compared to naïve populations in both CD4^+^ and CD8^+^ T cells (Fig. 3f). Stimulated CD4^+^ and CD8^+^ T_CM_ and CD8^+^ T_EM_ subsets also had higher frequency of cells with functional mitochondria than naïve populations (Fig. 3h). In contrast, a lower percentage of CD8^+^ T_EMRA_ had functional mitochondria and this number decreased when the cells were stimulated, which is consistent with previous studies showing mitochondrial impairment of this population^9,27^.

In a second assay, we performed the single cell energetic metabolism by profiling translation inhibition (SCENITH) assay that measures metabolism of individual cells based on relative translation rates and the incorporation of the puromycin into elongating ribosomes in the presence and absence of metabolic pathway inhibitors to assess glycolysis, OXPHOS, or fatty acid metabolism^28^ (Fig. 3i–p). The SCENITH data confirmed the Seahorse results showing that unstimulated CD4^+^ T_EM_ had higher glycolytic capacity than the naïve CD4^+^ T cells (Fig. 3i). In addition, all CD8^+^ MTC showed higher glycolytic capacity than the naïve CD8^+^ T cells. Glucose dependence; however, was significantly lower in only CD8 T_EMRA_ (Fig. 3j). Overall, unstimulated naïve T cells exhibited more mitochondrial dependence than the MTC (Fig. 3k). Together with the mitochondria staining data, these results suggest that the unstimulated MTC subsets have elevated OXPHOS metabolism compared to naïve populations, a finding that is also consistent with the RNA-seq data; however, resting naïve T cells depended on mitochondria more than the MTC subsets. CD8^+^ T_EM_ and T_EMRA_ cells exhibited an elevated fatty acid and amino acid oxidation capacity (FAO and AAO) compared to naïve CD8^+^ T cells (Fig. 3l). In general, when these cells were stimulated they reprogrammed their metabolism to be less dependent on mitochondria and instead increased glycolytic capacity to its maximum (Fig. 3m, n). We did not detect any significant difference in the metabolisms of stimulated naïve, T_CM_ and T_EM_ populations of CD4^+^ and CD8^+^ T cells (Fig. 3m–p). However, CD8^+^ T_EMRA_ cells showed less glycolytic capacity and glucose dependence, but higher mitochondrial dependence and FAO and AAO capacity than naïve CD8^+^ T cells. Taken together these data show the distinct transcriptional differences between naïve and MTC, the resulting functional consequences to cell metabolism and phenotype, and that these differences appear to increase in both number and magnitude as cells differentiate towards an effector-memory phenotype.

### The chromatin landscape of memory subsets correlates with transcriptional differentiation

**Fig. 4:**
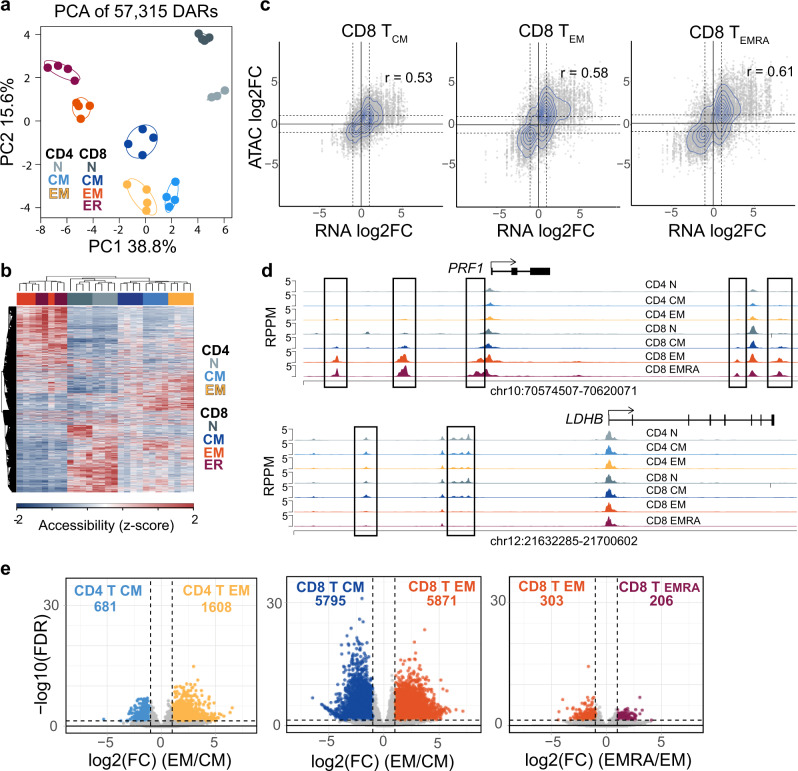
Effector-memory subsets exhibit a greater number of changes to chromatin accessibility compared to central memory subsets. **a** PCA plot of DAR from ATAC-seq data. **b** Heatmap displaying DAR z-score normalized across samples. **c** Scatter plots showing log_2_-fold changes (FC) of MTC subsets vs. naïve T cells of DEG (*x*-axis) mapped to DAR (*y*-axis) for CD8^+^ T_CM_, CD8^+^ T_EM_, and CD8^+^ T_EMRA_. Dotted lines represent ±1 log_2_FC. **d** Genome plots showing the average accessibility levels in each of the memory subsets and naïve T cells at the indicated locus. Data represent the mean of each cell type. DAR are highlighted by boxes. **e** Volcano plots showing DAR that are more accessible in CD4^+^ T_EM_ vs CD4^+^ T_CM_, CD8^+^ T_EM_ vs CD8^+^ T_CM_, and CD8^+^ T_EMRA_ vs CD8^+^ T_EM_. Number of DAR in each direction are totaled at the top of each plot.

To determine the extent to which the differences in gene expression were coordinated with changes to the chromatin organization in MTC subsets, the assay for transposase accessible chromatin-sequencing (ATAC-seq)^29^ was performed on the MTC subsets described above. This analysis resulted in the identification of 57,315 differentially accessible regions (DAR) between naïve, T_CM_, T_EM_, and T_EMRA_ cells of both CD4^+^ and CD8^+^ T-cell lineage groups (Supplemental Data 4). As with the RNA-seq data, principal component (PC) 1 separated effector-memory subsets (T_EM_, T_EMRA_) from T_CM_ and naïve T cells in a progressive manner (Fig. 4a). Hierarchical clustering of the DAR also revealed CD8^+^ effector-memory and naïve T cells as being the most distinct, with central memory and all CD4 MTC subsets sharing more similarity to naïve cells, and CD8 T_EM_/T_EMRA_ cells clustering closely together (Fig. 4b). Differential accessibility at regions that mapped to DEG was found to be positively correlated for each of the memory subsets (Fig. 4c, Supplemental Fig. 4a-b). Comparing the magnitude of these differences by fold change of both DEG and nearby DAR showed that gene expression and chromatin accessibility increased in both quantity and intensity in effector-memory cells compared to smaller differences from naïve T cells in central MTC (Fig. 4c). Some examples of DAR near DEG include the *PRF1* locus (Fig. 4d), which was found to have gained accessibility only in effector-memory subsets. Alternatively, chromatin at two regions upstream of the *LDHB* gene, which is downregulated in these subsets (Fig. 2j), was found to have decreased in accessibility in the effector-memory T cells (Fig. 4d). In a similar fashion, accessibility around gene loci expressed more highly in naïve or T_CM_ cells, such as genes encoding LEF1 and the G protein coupled receptor GPR15 (involved in T-cell homing), showed lower chromatin accessibility in T_EM_ subsets (Supplemental Fig. 4c and d). Overall CD4^+^ MTC exhibited relatively fewer DAR between T_CM_ and T_EM_ subsets compared to the large number of accessibility changes between CD8^+^ MTC subsets (Fig. 4e), and chromatin accessibility differences between CD8^+^ T_EM_ and terminally differentiated T_EMRA_ were the fewest in number (Fig. 4e). Collectively these data demonstrate that the transcriptional differentiation found in memory subsets is highly correlated with changes to chromatin accessibility.

### Differentiated chromatin between MTC subsets is enriched for bZIP, HMG, T-box, and bHLH transcription factor motifs

**Fig. 5:**
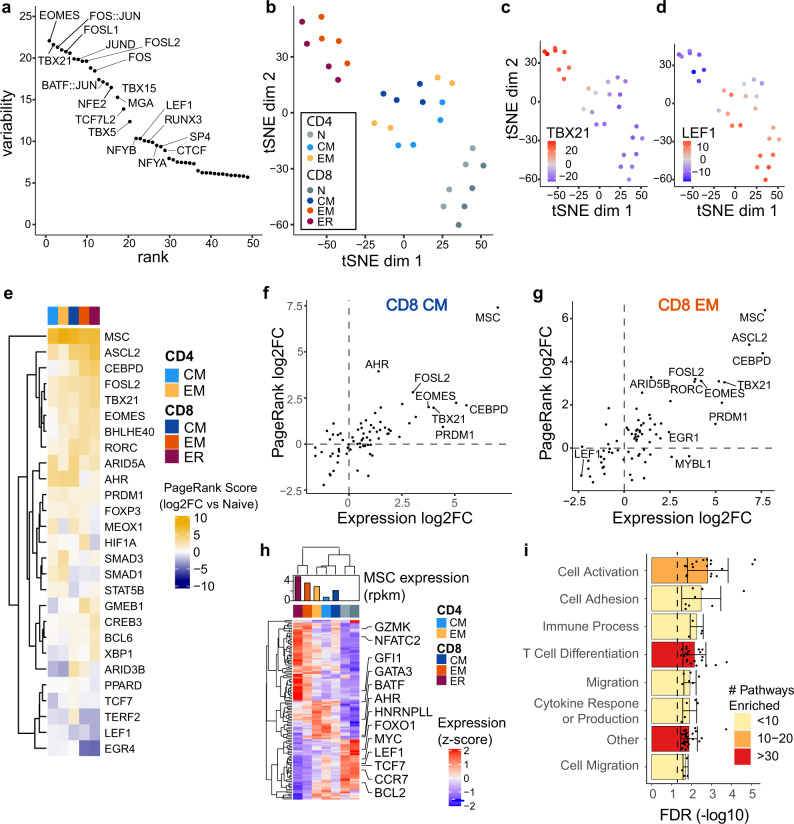
bZIP, HMG, T-box, and bHLH factors differentiate MTC subsets by chromatin accessibility. **a** Plot showing the top 50 transcription factors ranked (*x*-axis) according to variability (ChromVar) in chromatin accessibility (*y*-axis) around their corresponding binding motifs. **b** tSNE plot showing reduced dimensional visualization of variability of chromatin accessibility data for each memory-subset sample at all differential sites. **c,** **d** tSNE plot from B colored by ChromVar variability around *TBX21* (T-BET) and LEF1 binding motifs, respectively. **e** Heatmap of the log_2_-transformed fold change in PageRank statistics for each MTC subset (compared to naïve). **f,** **g** Scatter plots showing log_2_FC of RNA expression (*x*-axis) and PageRank statistic log_2_FC (*y*-axis) between memory and naïve samples for labeled CD8^+^ MTC subsets. **h** Heatmap of RNA expression from gene network of MSC transcription factor with bar plot showing expression of MSC in each subset. Data represent the mean expression of each cell subset. **i** Bar plot showing average gene set enrichment FDR corrected *p*-value from MSC network genes binned by T-cell-relevant categories. Error bars represent one standard deviation. Color corresponds to the number of pathways significantly enriched in each bin.

Transcription factors play key roles in orchestrating the global gene expression changes involved in establishing and maintaining cell type differentiation and establishing distinct gene regulatory states^30^. To identify potential transcription factors regulating differentiation of MTC subsets, we analyzed the variation of chromatin accessibility between subsets at sites with known binding motifs for transcription factors in the genome using chromVAR^31^. ChromVAR identifies motifs around which chromatin accessibility varies the most in a given set of samples (in this case cell subsets). We found that the chromatin accessibility around binding motifs for AP-1 (Jun, FOS, BATF), T-box (T-BET, EOMES), and HMG family transcription factors (LEF1) was the most highly variable across all MTC and naïve T cells (Fig. 5a). Visualization of the ChromVAR analysis data using tSNE projection distinguished samples by MTC subtype (Fig. 5b). Overlaying the ChromVar deviation score for individual transcription factor motifs onto the tSNE projection showed that T_EM_ cells had higher scores for T-box factors such as T-BET, encoded by *TBX21* (Fig. 5c), while naïve T cells and T_CM_ samples showed higher scores for LEF1 binding sites (Fig. 5d). Higher ChromVar scores were also found at AP-1 and IRF family binding motifs in the effector-memory samples (Supplemental Figs. 5a-f).

To further interrogate the various transcription factor regulatory networks involved in differentiating the MTC subsets the PageRank algorithm^32^ was used to correlate the presence of transcription factor binding motifs in accessible regions with changes in target gene expression, ultimately calculating a rank of transcription factor importance to the network. PageRank identified several transcription factors that increased in network rank in at least one MTC subset relative to naïve T cells and recapitulated naïve-associated transcription factors such as LEF1 and TCF7 (Fig. 5e). To compare subset differences, the fold change in PageRank scores from naïve cells was plotted against RNA expression for each factor in CD8^+^ T_CM_ and T_EM_ cells (Fig. 5f-g, Supplemental Fig. 5g-i). These data also corroborated the ChromVAR analysis by indicating both higher relative expression and PageRank scores for AP-1 and T-box factors, such as FOSL2, T-BET, and EOMES in the effector-memory subsets.

One transcription factor, MSC (encoding for musculin or ABF-1), was observed to have the highest rank score in all MTC subsets while being absent in naïve T cells. MSC has been previously identified as a transcriptional repressor capable of binding to E-box elements and implicated in the activation pathways of B cells, as well as the differentiation of peripheral CD4^+^ Treg cells^33,34^. Intracellular staining of MSC showed expression in all subsets with significant increases in stimulated T-cell subsets (Supplemental Fig. 6a), suggesting a potential role in T-cell activation. Analysis of the PageRank-generated regulatory network for MSC identified its potential for regulating many important genes in MTC subsets, including repression of LEF1 and TCF7 (Fig. 5h). The MSC target gene network was enriched for several key pathways such as cell activation, adhesion, cytokine production, and differentiation (Fig. 5i).

### bHLH Family Factors AHR and HIF1A potentially regulate different environmental responses in CD8^+^ T_CM_ and T_EM_ MTC subsets

**Fig. 6:**
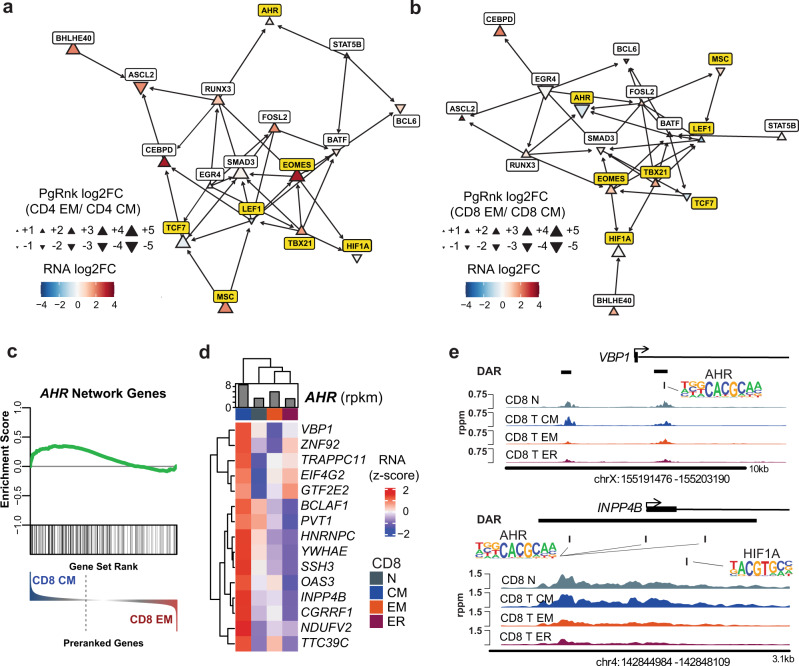
bHLH transcription factors AHR and HIF1A distinguish CD8 + T_CM_ and T_EM_. **a** Network diagram showing transcription factors in CD4^+^ MTC highly ranked by PageRank and associated connections to other TF. Triangles represent log_2_FC (size) and direction (orientation) of PageRank as indicated in the legend. Arrows represent putative regulation via nearby binding motifs. AHR, HIF1A, MSC, and other memory-subset defining TFs are highlighted in yellow. **b** Network diagram as in (**a**) for CD8^+^ T_CM_ and T_EM_. **c** GSEA plot using gene set made up of genes putatively regulated by AHR showing enrichment in CD8^+^ T_CM_ vs. T_EM_ DEG. **d** Heatmap of leading-edge genes from GSEA most highly upregulated in T_CM_. Bar plot indicates normalized expression of AHR in rpkm. Data represent the mean expression of each cell subset. **e** Genome plots showing the average accessibility levels in each of the CD8^+^ memory and naïve T cells at the indicated locus. DAR are highlighted by black horizontal bars, with AHR and HIF1A binding motifs at indicated locations. Data represent the mean of each cell type.

Reconstructing transcription factor regulatory networks via PageRank-predicted interactions emphasized the centrality of key transcription factors LEF1, TCF7, T-BET, and EOMES, as well as differences in TF importance by subset. MSC was predicted by PageRank to regulate LEF1 and TCF7, potentially leading to higher repression of these factors in CD4 + T_EM_ and other effector-memory subsets (Fig. 6a). The bHLH transcription factor AHR was highly induced as seen by intracellular protein staining in all T-cell subsets (Supplemental Fig. 6a). Intriguingly, AHR also showed both high relative expression and PageRank score exclusively in the CD8^+^ T_CM_ subset, while bHLH transcription factor, HIF1A (hypoxia-inducible factor 1α), was more highly ranked by PageRank in CD8^+^ T_EM_ cells (Fig. 6b, Supplemental Fig. 5k). AHR protein levels were increased in all subsets upon stimulation. HIF1A was increased after stimulation in all T-cell subsets except for T_EM_, which maintained more constant concentrations of this protein (Supplemental Fig. 6a). AHR has been implicated in sensing xenobiotics and T-cell homing to tissues^35^, while HIF1A has previously been associated with sensing hypoxic conditions and modulating metabolism in these circumstances^36^. HIF1A and AHR are known to have an antagonistic relationships with respect to target genes and their own expression^37,38^. GSEA using the list of genes putatively regulated by AHR showed significant enrichment of these genes in those upregulated in CD8^+^ T_CM_ vs CD8^+^ T_EM_ (Fig. 6c). Expression of several of these genes in the leading edge of GSEA enrichment were positively correlated with AHR expression in MTC and were exclusively expressed in T_CM_ (Fig. 6d). Two of these genes, *VBP1* and *PVT1*, along with *HIF1A* are part of the hypoxia response^39,40^. Two DAR with greater accessibility in T_CM_, were found near the promoter region of the *VBP1* gene, one of which contains a binding motif known to bind AHR (Fig. 6e). *VBP1* encodes for the protein VHL which is the substrate recognition subunit of an E3 ligase known to target HIF1A for degradation^39^. Another DAR with higher accessibility in T_CM_ and naïve T cells was found at the promoter of the *INPP4B* gene, encoding inositol polyphosphate-4-phosphatase type II B (Fig. 6e). This region contains three AHR binding motifs as well as a motif specific for HIF1A binding, suggesting potentially competitive regulation between these two factors. In total, these data suggest that bZIP, HMG, and T-box family transcription factors are important for memory-subset differentiation, and additionally bHLH family factors, such as MSC, AHR, and HIF1A may play key roles in regulating distinct subsets and their transcriptional programs before and following stimulation.

### Genes that are uniquely upregulated in stimulated memory-subset cells include Induced and Augmented transcripts

**Fig. 7:**
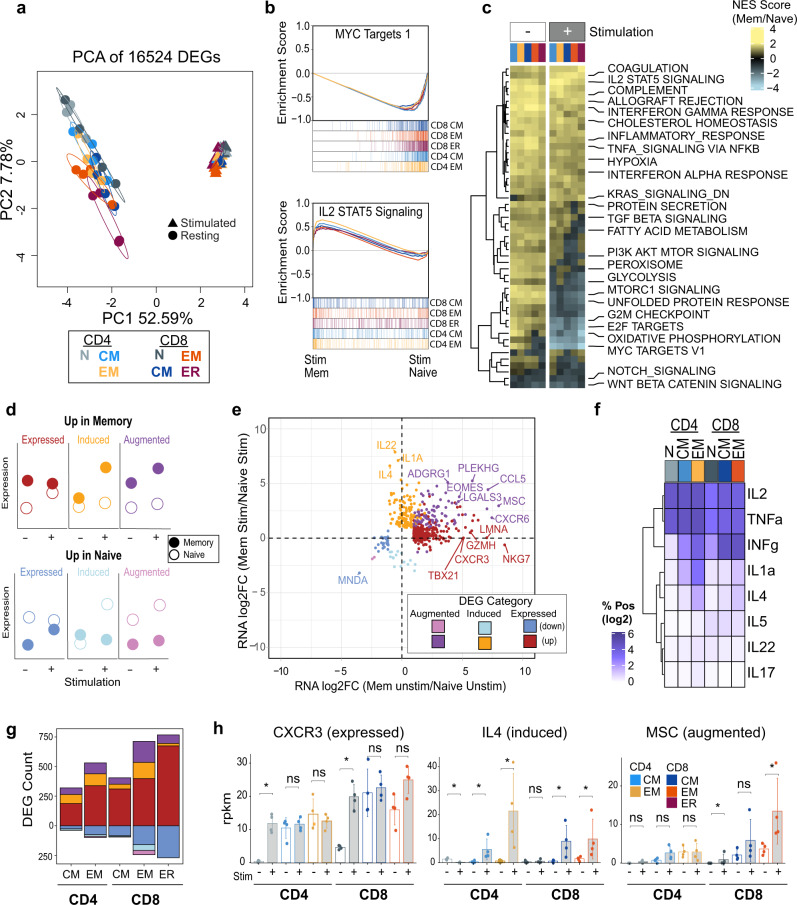
MTC induce or augment unique gene expression programs after stimulation. **a** PC analysis plot of DEG for resting (circle) and stimulated (triangle) memory and naïve T-cell samples. **b** GSEA plots for indicated gene sets showing the enrichment score for each stimulated MTC subset as compared to stimulated naïve T cells. **c** Heatmap of normalized enrichment scores (NES) from GSEA of both stimulated and unstimulated MTC compared to respective naïve T cells. **d** Schematic describing examples of DEG categorization; before and after stimulation (Stim). **e** Scatter plot showing log_2_FC of DEG in unstimulated vs. stimulated MTC. Dots are colored according to DEG category as indicated. **f** Heatmap showing log_2_ transformation of the percent of cells positive for respective cytokine protein expression. **g** Bar plot showing number of DEG compared to naïve T cells belonging to Expressed, Induced, or Augmented categories for each memory subset. **h** Gene expression bar plots showing rpkm for indicated genes. Error bars represent ±1 SD from mean in each group. Asterisks indicate relevant significant differences as detected by DESeq2 algorithm.

To better understand the transcriptional and epigenetic properties that allow memory T-cell subsets to rapidly respond to stimulation, all five MTC subsets, as well as naïve T cells were stimulated using anti-CD3/CD28 beads ex vivo for 42–48 h. RNA-seq analysis of the stimulated T cells showed a profound change in transcriptional programs after stimulation and much of this response was homogeneous across the different MTC subsets. This is highlighted by PCA wherein PC1 (52.59% variation) separated resting cells from all those that have been activated (Fig. 7a). GSEA showed that activated naïve cells more strongly upregulated pathways such as MYC target genes to a greater extent than in MTC, while activated MTC more strongly upregulated genes associated with IL-2, STAT5 signaling, and other immune response signaling pathways (Fig. 7b). Despite a large degree of consistency in response to stimulation in the different memory subsets, gene sets related to fatty acid metabolism, glycolysis, and MTOR signaling were enriched to varying degrees in GSEA between T_CM_ and T_EM_ cell subsets after stimulation (Fig. 7c).

To identify genes that were differentially expressed upon stimulation in MTC subsets, expression patterns were examined that were specific to MTC. Overall, the data revealed the presence of three main groups of genes that change in expression compared to naïve T cells before and after stimulation as hypothetically illustrated in Fig. 7d. Each group has two expression states: (a) its constitutive expression in unstimulated MTC vs. naïve; and (b) its change in expression following stimulation. Expressed genes had high fold-change differential expression between naïve and MTC, which naïve T cells induced only after stimulation. Induced genes were similarly expressed in both resting naïve and MTC and are induced in only the MTC group following stimulation. Augmented genes showed expression differences between MTC and naïve cells in both the resting and stimulated states (Fig. 7d). Overlaying these categories on gene expression differences of naïve versus memory resting and stimulated T cells showed that the majority of such DEG were positively upregulated in MTC (Fig. 7e). The memory Induced gene category was highly enriched for cytokine genes including *IL4* (Fig. 7h), *IL1A*, and *IL22*. These genes were uniquely expressed by MTC subsets to varying degrees after stimulation but repressed in activated naïve T cells at 48 h after the same stimulation. To confirm these results, we measured a panel of common cytokines using intracellular staining coupled with flow cytometry (Supplemental Fig. 7). Some cytokines, such as IL-2 and TNFα were expressed in a significant number of cells by all activated T cells studied. In contrast, the production of effector T-cell-associated cytokines (IFNγ, IL-1α, IL-4, IL-17, and IL-5) was differentially increased in various MTC after stimulation compared to stimulated naïve T cells (Fig. 7f). Il-22 protein was significantly induced in naïve and T_EM_ CD4^+^ MTC. The T_EM_ MTC subsets of both CD4^+^ and CD8^+^ lineage produced the highest level of cytokines after stimulation in comparison to naïve or T_CM_ cells (Supplemental Fig. 7). The stimulation-independent Expressed gene category contained the most DEG overall, but each memory subset also exhibited Induced and Augmented genes (Fig.7g). Genes found within the Expressed category for T_EM_ cells included important effector T-cell molecules, such as granzyme H, encoded by *GZMH*; T-BET; and *CXCR3*, a chemokine receptor associated with T_H1_ CD4^+^ T cells and effector CD8^+^ T cells^41^ (Fig. 7h). Both CD8^+^ and CD4^+^ T_EM_ cells showed higher numbers of Induced and Augmented genes than T_CM_. Intriguingly, *MSC* was also found to exhibit an Augmented expression pattern in these cells after stimulation, while remaining repressed in stimulated naïve T cells (Fig. 7h). Thus, despite both MTC and naïve T cells activating several similar gene programs upon TCR stimulation, MTC subsets have unique induction/expression profiles upon stimulation, a property that may provide them with a greater degree of efficiency and capacity upon rechallenge.

### Augmented gene expression is correlated with epigenetic changes introduced by earlier activation of naïve cells

**Fig. 8:**
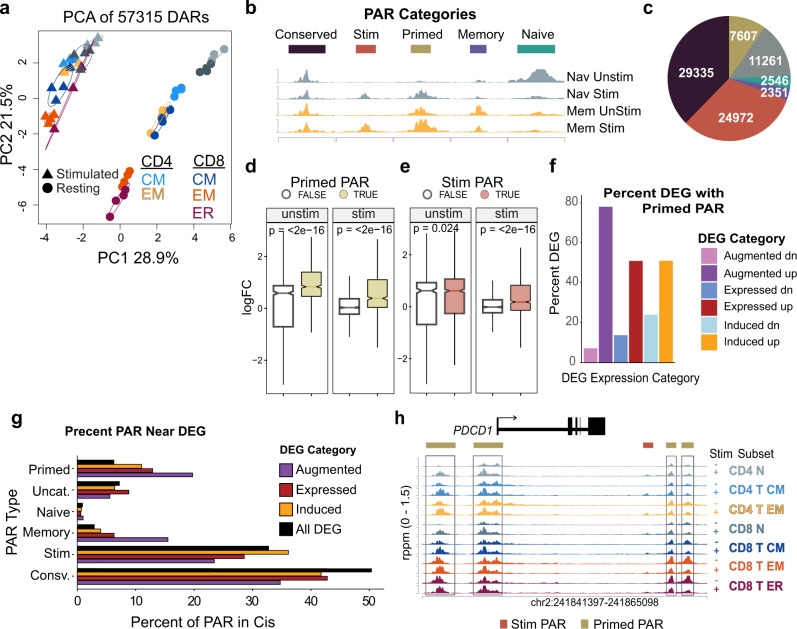
Accessible chromatin regions primed from previous activation are associated with augmented gene expression of MTC. **a** PC analysis plot of DAR between unstimulated and stimulated memory and naïve T-cell samples. **b** Schematic illustrating examples of PAR categorized by accessibility in both unstimulated (Unstim) and stimulated (Stim) states in memory (Mem) and naïve (Nav) T cells. **c** Pie chart showing number of DAR peaks in each category. **d** Box plot showing log_2_FC of DEG found to be significant in either unstimulated memory vs unstimulated naïve T cells or stimulated memory vs stimulated naïve T cells. Boxes colored in gold represent log_2_FC distribution of genes with at least one primed-PAR mapped to a differential gene locus. Notches represent median values, box limits represent upper and lower quanrtiles. Lines represent range. **e** Box plot as in (**d**). Boxes colored in red represent log_2_FC distribution of genes with at least one stimulation-PAR mapped to a differential gene locus. **f** Bar plot showing the percent of genes belonging to either expressed, induced, or augmented expression categories with at least one primed-PAR nearby. **g** Bar plot showing the proportion of each PAR type compared to all peaks surrounding DEG belonging to each of the different memory-specific expression categories. The *All DEG* category represents all genes differentially expressed compared to naïve T cells. **h** Genome plot showing accessibility data around the *PDCD1* locus. DAR are indicated by horizontal bars colored according to PAR category above tracks. Primed-PAR are highlighted by boxes.

To determine the of role chromatin accessibility in the unique response to stimulation described above, ATAC-seq was performed on the stimulated memory and naïve T cells. Similar to the global transcriptional response to stimulation, PCA of the ATAC-seq datasets showed that the greatest amount of variation separated stimulated from unstimulated cells, and that differences between memory subsets were diminished in the stimulated samples (Fig. 8a). Individual peaks of accessibility in the ATAC-seq analysis followed a set of a patterns that we have termed patterned accessibility regions (PAR) (Supplemental Data 5). PAR are defined by chromatin state before and after stimulation. Five categories of PAR emerged from the analysis: conserved, stimulated, primed, memory, and naïve (Fig. 8b). Conserved-PAR were unchanged across all subsets and stimulation states, whereas stimulation-PAR were present only after stimulation. Each of these two PAR groups occurred equally in both naïve and MTC and made up the majority of the accessible chromatin regions found in the ATAC-seq data in all memory subsets (Fig. 8c, Supplemental Fig. 8a). Conserved-PAR were also found to be more highly enriched in promoter regions than other PAR categories (Supplemental Fig. 8b). PAR specific to MTC occurred in one of three ways: memory-PAR were specific to MTC and were unchanged by stimulation; naïve-PAR were present in resting naïve T cells, but neither their stimulated counterparts nor in MTC; and finally primed-PAR were accessible in resting and stimulated MTC and became accessible upon activation in naïve T cells. Primed-PAR were more than twice as abundant as memory- or naïve-PAR.

In examining the relationships between PAR types and gene expression, we observed that the presence of at least one primed-PAR was strongly correlated with upregulation of DEG in MTC for both resting and stimulated states (Fig. 8d). A similar influence of stimulated-PAR was found in the stimulated but not the resting MTC transcriptional data (Fig. 8e). The presence of memory-PAR was found to be correlated with both resting and stimulated expression in MTC, while the presence of naïve-PAR biased gene expression in most nearby genes towards higher expression in naïve T cells (Supplemental Figs. 9a, b). Comparing these nearby DEG to the MTC-specific gene programs as defined by unique expression after stimulation (Expressed, Augmented, or Induced) revealed that nearly 80% of the upregulated genes in the augmented category had at least one primed peak in *cis* (Fig. 8f). Additionally, compared to the genome-wide prevalence, primed- and memory-PAR made up a significantly higher proportion of all the accessible regions surrounding DEG that were augmented in MTC (Fig. 8g). This is exemplified by the *PDCD1* locus, a gene showing augmented expression in MTC, which is also composed primarily with primed-PAR (Fig. 8h). These data support an epigenetic mechanism of enhanced recall in which primed-PAR are inscribed in the epigenome upon previous activation of naïve T cells^42^ and occur within regions of the genome that are associated with higher expression levels of important genes in MTC.

### PAR contain distinct sets of transcription factor motifs that segregate T cells by lineage and memory subtype

**Fig. 9:**
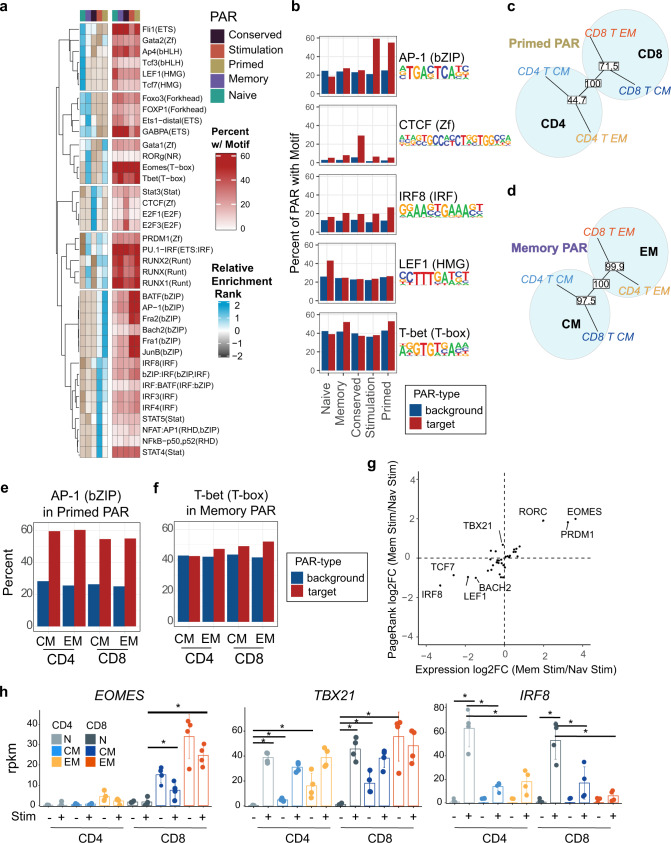
Transcription factor motif and expression patterns differentiate between memory and naïve T-cell response to stimulation. **a** Heatmaps of enrichment rankings for transcription factor binding motifs in each of the PAR categories for CD8^+^ T_EM_ cells and percent of PAR with the target motif for the selected example motifs. **b** Bar plots showing percent of input peaks containing indicated motifs (red) and percent background regions containing motif (blue) for five motifs representing enrichment specific to one or more of the PAR categories. **c,** **d** Phylogenetic dendrogram of transcription factor binding motif enrichment ranks found within Primed (**c**) and Memory (**d**) PAR compared across MTC subset groups. The reproducibility of tree structures was tested using bootstrapping analysis with the percent reproducibility shown in white boxes for each node. **e,** **f** Bar plots showing examples of differences in percent input PAR containing the indicated motifs across MTC subsets for primed (**e**) and memory (**f**) PAR. **g** Scatter plot showing log_2_FC of RNA expression (*x*-axis) vs PageRank statistic (*y*-axis) of CD8^+^ T_EM_ compared to naïve T cells for transcription factors known to bind to motifs found to be enriched in primed or memory-PAR. **h** Bar plot showing rpkm values for indicated genes in both resting and stimulated samples. Error bars indicate ±SD. Asterisks indicate relevant significant differences as detected by DESeq2 algorithm.

To identify transcription factors associated with each of the identified PAR categories, motif discovery, and enrichment analysis was performed using HOMER^43^. To control for uneven power, driven by unequal numbers of individual peaks in each category (Fig. 8c), enriched transcription factor binding motifs in DAR were compared across all five peak categories by relative ranking of enrichment *p*-values (Fig. 9a). Enrichments of HMG family transcription factors (LEF1 and TCF7) were more highly ranked within naïve-PAR, and CTCF-motif enrichment was ranked highest in the constitutively accessible conserved-PAR (Fig. 9b). Interestingly, several transcription factor motifs were enriched in both primed- and stimulation-PAR, including the AP-1 family factors (Fig. 9b). Motifs specific for other transcription factors were enriched in both primed-and memory-PAR, including the T-box factors EOMES and T-BET.

Enrichment rank scores of transcription factor motifs associated with primed-PAR across the different memory subsets in CD8^+^ and CD4^+^ T cells clustered according to cell lineage (Fig. 9c); whereas transcription factor motifs enriched in memory-PAR clustered according to memory cell subtype (Fig. 9d). Differences between memory subsets were also observed, with CD4^+^ T cells showing slightly higher percentages of primed-PAR containing AP-1 factor motifs (Fig. 9e). The percentage of memory-PAR containing T-BET motifs was highest in CD8^+^ T_EM_ cells and lowest in CD4^+^ T_CM_, a finding that is consistent with the role of T-box factors in effector T-cell programming (Fig. 9f)^44^. The PageRank algorithm was used to compare the relative importance of different transcription factors in terms of their effect on target DEG after stimulation. EOMES was found to have the highest PageRank score in stimulated CD8^+^ MTC (compared to stimulated naïve), as well as the greatest fold change in augmented gene expression (Fig. 9g, i), a finding consistent with its role in maintaining CD8^+^ T-cell memory^44,45^. PageRank scores for EOMES and T-BET were similar. T-BET expression was lowest in naïve cells but varied across MTC subsets and was induced to similar levels irrespective of subset (Fig. 9h). Protein measurements of both intracellular EOMES and T-BET via flow cytometry showed that each were increased after stimulation (Supplemental Fig. 6a); however, EOMES levels were already significantly high in resting CD8 T_EM_ and remained unchanged at these high levels after stimulation. T-BET levels were induced to the highest degree in naïve T cells in both CD4 + and CD8 + lineage groups. Likewise, the PageRank score and expression of *IRF8* were highest in stimulated naïve T cells (Fig. 9g). Expression data showed that stimulation induced high levels of *IRF8* in naïve cells, but all memory subsets induced significantly lower levels of *IRF8* after the 48 h of stimulation (Fig. 9h) compared to naïve T cells. Collectively these data show that both distinct and overlapping sets of transcription factor binding motifs are enriched in each of the identified PAR categories. Moreover, the expression of many of the transcription factors known to bind such motifs (IRF8, EOMES, T-BET, and LEF1) are coordinated to regulate and enable unique aspects of MTC differentiation following stimulation.

## Discussion

In this study we used an integrated transcriptomic and epigenetic sequencing approach to understand the cellular programming of the largest memory-subset groups found in human blood and how these programs change in response to ex vivo stimulation. In addition to specific and differential relationships between subsets and lineages, a series of regulatory modalities, pathways, and transcription factors were found to be associated with specific MTC genes. States of chromatin accessibility in key genes within MTC revealed a robust mechanism to control gene expression in response to secondary activation that allow MTC to respond more efficiently and with fewer epigenetic reprogramming steps. Regardless of cell lineage, MTC subsets shared nearly one third of the DEGs that distinguish them from naïve T cells, suggesting a common memory signature. However, expression differences between each MTC subset and naïve T cells were also observed in gene pathways related to important functions such as cytotoxicity, metabolism, and self-renewal.

It is known that MTC exploit distinct metabolic pathways based on their differentiation and memory status^46–48^. Here, in addition to the transcriptional evidence, we provided additional characterization of the metabolic states of naïve and MTC subsets. Consistent with previous studies^49^, we showed that resting MTC populations predominantly utilize OXPHOS and fatty acid oxidation for their main or primary metabolic needs and switch their metabolism to aerobic glycolysis when they are stimulated ex vivo. In addition, some of the MTC subsets use the other pathways to different degrees as a secondary means of metabolism. Previous studies revealed that although both resting naïve and MTC rely on OXPHOS, naïve T cells harbor less mitochondrial mass^50^. In addition, mitochondria of MTC display more elongated structures with tight cristae compared to naïve counterparts, indicating that OXPHOS is highly efficient in these cells^51,52^. Supporting these studies, our mitochondria analysis of T-cell subsets demonstrates distinct distribution of populations with MTG^Hi^ and TMRM^Hi^ among the subsets according to their activation state. In particular, T_EM_ and T_CM_ subsets exhibit significantly higher frequency of cells with greater mitochondria mass and potential than naïve T cells. By contrast, CD8^+^ T_EMRA_ cells did not show a significant difference compared to their naïve counterpart, and stimulation of these cells further decreased the frequency of MTG^Hi^ and TMRM^Hi^, suggesting that these cells are metabolically distinct than the other MTC subsets. CD4^+^ MTC subsets have a similar fatty acid oxidation capacity; however, CD8^+^ T_EM_ and T_EMRA_ subsets show increased capacity for this metabolic pathway compared to naïve counterparts. Consistent with previous studies^53^, all activated T cells decreased fatty acid utilization.

Recent studies showed that MTC shift towards aerobic glycolysis more rapidly than naïve T cells to facilitate a rapid secondary response^54^. We showed that T_EM_ and T_CM_ subsets differ in expression of genes related to the regulation of metabolism and in particular to acetyl CoA production with differential expression of glycolysis-promoting enzymes such as LDH. Our metabolic data confirm that all resting MTC subsets have higher glycolytic capacity than their naïve counterparts, suggesting a potential contribution to more efficient secondary responses. Moreover, the glycolytic capacity of the effector MTC is significantly higher than central memory and naïve subsets that might be required for their rapid effector function.

Overall, our data show evidence of a gradient of differentiation from naïve T-cell progenitors in MTC subsets which is highest in T_EM_. We show that this subset exhibits the greatest numbers of changes from the naïve T-cell transcriptome as well as higher numbers of changes to chromatin accessibility. T_CM_, on the other hand, maintained the highest expression levels of genes that were also expressed in naïve T cells, a result consistent with other recent findings^55^. In addition shared expression of genes in naïve T cells and T_CM_ MTC includes the transcription factors downstream of the WNT-beta catenin signaling pathway (LEF1 and TCF1), which have been previously associated with self-renewal and stem-like properties in mature CD8^+^ T cells^56^. It is important to note that the naïve CCR7 + CD45RA + T cells isolated in this study may also contain a small percent of stem-cell MTC: however, this population has been shown to make up less than 10% of total CD8^+^ T cells and so likely had a small effect on our data^57^.

One mechanism by which MTC may be able to alter the way in which they respond to antigen stimulation compared to naïve T cells is through modification of chromatin structure. Large numbers of chromatin accessibility and epigenetic modifications such as DNA methylation changes have been observed previously in mouse CD8^+^ MTC after acute or chronic viral infections^13,58^. Here, we found that all human CD8^+^ and CD4^+^ MTC contain large numbers of DAR compared to naïve T cells. These DAR fell within loci that were correlated with differential gene expression, and which progressively increased in both number and magnitude from central memory to effector MTC subsets. It remains unclear whether this variation in epigenetic changes between central and effector MTC reflects a one-way linear differentiation path in CD8^+^ T cells as previously suggested by some models^59^, or results from a more complicated plastic development trajectory^58,60^. However, fewer differences were found when comparing the chromatin of CD8^+^ T_EM_ to T_EMRA_, suggesting a closely shared differentiation pathway for these cells. Interestingly, accessibility differences between circulating CD4^+^ T_CM_ and T_EM_ were subtle, reflecting their close relationship. This may reflect a heterogenous population not as well captured by traditional T_CM_ and T_EM_ definitions, as the diverse roles CD4^+^ effector T cells play in the immune response is likely to lead to a memory population that is equally as diverse. Upon activation, however, CD4^+^ T_EM_ show a greater degree of transcription and chromatin accessibility changes (greater than CD4^+^ T_CM_), which may be related to the activation requirement of effector functions of different CD4^+^ T-cell effector subsets.

Transcription factor motifs associated with DAR and DEG identified sets of potential factors that could be responsible for defining MTC transcriptional networks. T-box, AP-1, and HMG family transcription factor binding motifs were enriched within DAR that separate memory from naïve T cells, particularly those that distinguish effector memory. Supporting these results, the T-box factors T-BET and EOMES are known to be important in the formation of CD8^+^ effector T-cell programming and memory cell populations respectively^44,61^. The HMG factor LEF1 has also been implicated in early T-cell differentiation^62^. Members of the bHLH family of transcription factors, such as AHR, HIF1A, and MSC were found to be important for distinguishing between the memory subsets. AHR and HIF1A are capable of sensing changes in environmental conditions^38,63^. An AHR binding motif found within a DAR near the promoter of *VBP1* (encoding VHL) suggests one potential mechanism by which AHR modulates metabolic differences of CD8^+^ T_CM_ and T_EM_, as conditional deletion of this gene has been shown to promote constitutive glycolysis in CD8^+^ T cells reminiscent of that exhibited by T_EM_^64^. Our data suggests that AHR plays a greater a role in the CD4^+^ MTC and CD8^+^ T_CM_ subsets, including the highest protein expression of AHR in T_CM_ after stimulation. The integrated assay data also suggested that HIF1A might be more important in the CD8^+^ T_EM_ population. However, interestingly, protein data for this factor showed that T_EM_ populations were the only subset studied for which HIF1A concentration did not increase after stimulation. Ultimately other mechanisms (such as dynamic degradation by AHR-regulated genes like *VBP1* (VHL)) could be a part of differential environmental sensing mechanisms between MTC subsets^65^. The evidence for unique regulation of HIF1A and its targets in T_EM_ shown here suggests a potentially important role in this MTC subset which will require further experimentation to elucidate.

MSC was the most highly ranked transcription factor in all MTC by the PageRank algorithm, but its expression was absent in naïve T cells. MSC is a repressor capable of binding E-box elements as either a homodimer or heterodimer with E2A (TCF3) and was initially found to be highly expressed in activated B cells where it has been shown to play a role in promoting memory B cell differentiation^33,66^. We showed that it is also highly expressed in activated T cells as well. The potential interactions of MSC with E2A is particularly interesting in the context of memory T cells as motifs specific for this factor were found to surround several genes repressed in activated CD8^+^ T cells in mice^13^. MSC is expressed by several CD4^+^ effector T-cell subsets, including Tfh, Th17, and Treg cells in mice^34,67^. In human Th17 cells, MSC inhibits cellular response to IL-2 via STAT5B signaling^68^. Studies using genetic mouse models have shown that MSC deficiency leads to spontaneous gut and lung inflammation with age^34^ while also enhancing inflammation and IL-22 secretion in inflammatory bowel disease models^69^. Despite its clear role in a variety of settings and evidence of its importance for MTC gene regulatory networks shown here, it is largely unclear exactly how MSC participates in regulating MTC, and what role it plays in activation of naïve and MTC respectively.

Stimulation of T cells led to dramatic changes in both gene expression and chromatin accessibility. Various cytokines genes (*IL4, IL1A,* and *IL22*) were induced after 48 h of stimulation in MTC but not in naïve T cells, potentially reflecting a mechanism for expediting effector gene expression that is unique to antigen-experienced cells. We showed that induction of these cytokine transcripts translates directly to higher levels of cytokine protein in MTC relative to naïve cells under similar stimulation conditions. This effect is most prominent in T_EM_ subsets of both CD4 + and CD8 + MTC which express the highest levels of their respective effector cytokines. MTC also constitutively express a large number of genes that are not expressed in naïve T cells but are upregulated following stimulation. Continuous expression of effector genes in this category reflects the ability of MTC to maintain certain effector functions, even in the absence of active infection, as exemplified by increased expression of the gene *CXCR3* known to play a role in trafficking CD4^+^ and CD8^+^ T cells to peripheral sites of inflammation^41^. Several MTC-specific genes were augmented in their expression, having high levels in the resting state that were increased further following stimulation. These genes were correlated with accessible chromatin loci that were present only in MTC prior to stimulation, suggesting that these genes were poised for expression upon rechallenge. The transcription factor EOMES exemplifies this set, with higher expression in MTC potentially facilitating its central role in the maintenance/programming of these cells^44,70^.

Binding motifs specific for known activation-induced transcription factors such as AP-1 family members (e.g., BATF) and their binding partners NFAT and IRF were highly enriched in both stimulation- and primed-PAR, suggesting that these regions may also be maintained in an open state in MTC in order to more rapidly respond to TCR stimulation. Binding site motifs for transcription factors at primed-PAR separated the samples by lineage (CD4^+^ vs CD8^+^) suggesting distinct epigenetic control of activation for each lineage. Interestingly, binding motifs within memory-PAR separated the samples by subset (T_CM_ vs T_EM_) irrespective of lineage. The exact cues leading to specific MTC subset differentiation remain elusive; however, it is likely that epigenetic mechanisms targeting these memory-subset-specific PAR play a role when paired with simultaneous expression of master regulator transcription factors.

Transcription factor motifs may play multiple roles depending on whether they are maintained in an accessible state within naïve or MTC, and when matched with expression of their corresponding TF. For example, in all MTC, IRF8 motifs are enriched in primed- and stimulation-PAR, but network analysis suggests that this factor plays a greater role in expression changes of activated naïve T cells than in activated MTC, due perhaps to its lowered expression in MTC. Coupled with IRF8’s known role in driving effector T-cell generation in mice^71,72^, this may suggest repression of IRF8 as an important aspect of T-cell memory. Conversely, as noted above, the expression of EOMES is augmented in CD8^+^ MTC before and after stimulation while expression of the competing factor T-BET is similar between naïve and MTC after stimulation. Expression of EOMES after stimulation in CD8^+^ MTC is slightly decreased after stimulation, but still expressed to a greater extent than in stimulated naïve cells, perhaps leading to a higher overall ratio of EOMES/T-BET expression in stimulated MTC. This along with the presence of EOMES/T-BET binding motifs within both memory- and primed-PAR highlights a potential mechanism by which EOMES helps drive memory-specific gene expression, particularly in CD8 + T_EM_ cells, which express high levels of this factor in a resting state. In its absence, T-BET controls the effector cell transcriptional response after activation^61^. Accordingly, higher resting expression of T-BET and greater accessibility of its target sites in T_EM_ may drive the effector-like phenotype of this subset.

In summary, MTC display lineage and subset-specific gene expression and chromatin accessibility patterns. These provide MTC with a unique epigenetic context driven by a history of their previous activation during encounters with antigen. In total, these memory-specific features enable MTC to adopt expression profiles of effector T cells more rapidly during secondary immune challenge.

## Methods

### Human subjects

Whole blood samples from four deidentified individuals were obtained with informed consent in accordance with Emory University School of Medicine Institutional Review Board protocols, IRB00045821. PBMCs were separated by density gradient centrifugation (Ficoll-Paque, GE Healthcare), treated with ACK lysing buffer to remove red blood cells, and washed in PBS.

### MACS isolation and ex vivo stimulation

CD4^+^ or CD8^+^ T cells were isolated from PBMC samples using MACS microbead (Miltenyi Biotec) isolation kits (CD4: #130-096-533, CD8: #130-096-495) via negative selection of non-target cells. Briefly, 1 × 10^7^ cells were resuspended in 40 μl MACS buffer and incubated with CD4^+^ or CD8^+^ biotin-antibody cocktail and then incubated with 20 μL of T-cell MicroBead cocktail for 10 min. The flow-through was collected from a MACS separator column and washed with 3 mL MACS buffer. Collected cells were separated for either immediate flow sorting (resting cells) or incubated for 42–48 h in complete Roswell Park Memorial Institute (RPMI) media with anti-CD3/CD28 beads (Gibco: #11131D) for ex vivo stimulation. 10^6^ cells were added to cell suspensions in a 2:1 bead-to-cell ratio before being removed prior to flow staining and sorting.

### Flow cytometry isolation of human memory T-cell subsets

Cells were resuspended at 1 × 10^6^/100 µl in FACS buffer (PBS, 1% BSA, and 2 mM EDTA), stained with CCR7-BB515 (BD biosciences: 565870) for 30 min at 37 °C, and then a cocktail of the following: CD3-V450 (Tonbo Biosciences: 75-0038), CD4-PE/Cy7 (Biolegend; 300511), CD8-FITC (Life technologies: MHCD08014), CD45RA-PE/TxRed (Biolegend: 304145), Zombie Yellow Fixable Viability Kit (Biolegend; 423104)) for 30 min at 4 °C and then washed with 1 ml of FACS buffer. The following gating strategy was used to define memory subsets: lymphocytes were gated based on SSC-A / FSC-A, single cells by FSC-H / FSC-A, and live cells were based on exclusion of Zombie Yellow Fixable Viability Kit. T cells of the appropriate linage were selected using the markers CD3, CD4, and CD8. Memory and naïve T-cell subsets were isolated using the markers CCR7 and CD45RA. Cell sorting was performed at the Emory Flow Cytometry Core using a FACSAria II (BD Biosciences) and BD FACSDiva software (BD Biosciences). Data were analyzed and figures generated using FlowJo v10.6.2. Supplementary Table 1 contains a list of all antibodies and the concentrations used.

### RNA sequencing

One thousand cells were sorted directly into RLT buffer (79216; Qiagen) containing 1% 2-mercaptoethanol. RNA was isolated using the Quick-RNA Microprep kit (Zymo Research; R1050). The SMART-Seq v4 Ultra Low Input RNA Kit (634894; Takara Bio) was used for cDNA synthesis, and 400 pg of cDNA was used as input for the NexteraXT kit (Illumina) to create sequence libraries. DNA libraries were sequenced at the University of Alabama at Birmingham’s Heflin center for genomics using a NextSeq500.

### RNA-sequencing data analysis

Raw sequencing data were mapped to hg38 using STAR v.2.5.3^73^. Duplicate reads were identified and removed using PICARD (http://broadinstitute.github.io/picard/). Reads per kilobase per million (rpkm) normalized gene expression counts were derived by analyzing coverage across all exons that fall within unique ENTREZ genes using the GenomicRanges package^74^. The Bioconductor package DESeq2^75^ was used to determine differentially expressed genes (DEG) which were defined as having an absolute log_2_ fold-change of ≥1 and a false discovery rate (FDR) of ≤0.05. Differentially expressed genes are listed in Supplemental Data 1. All detected transcripts were pre-ranked for gene set enrichment analysis (GSEA)^76^ by multiplying the sign of the fold change (±) by −log_10_ of the *p*-value. Heatmaps were generated using the CompexHeatmap R package^77^. Three dimensional comparisons of differential expression used the normalized read counts which were averaged across MTC subset groups, log_2_ transformed, and converted to barycentric coordinates for visualizing using the Tri-wise R package^20^. T-cell-relevant gene sets were obtained from the MSigDB v7.4 database gene ontology collection (GOBP-GO _ NATURAL _KILLER_ CELL_MEDIATED_IMMUNITY; GOBP-LEUKOCYTE_MEDIATED_CYTOTOXICITY), or derived by taking the top 100 upregulated genes in CD8 CD101^—^,Tim3^—^ cells as described by Hudson, et al. ^21^. Gene modules were discovered by analyzing genes with detected counts, filtering for DEG, and then filtering for genes which had an expression value ≥3 rpkm across all samples in any one sample group. Clustering was performed using fuzzy c-means clustering (using the ‘cmeans’ function of the e1071 R package) after first estimating the fuzzifier parameter^78^ and a selecting a c value based on analysis of within sum of squared error. Module assignments for each gene are listed in Supplemental Data 2 (CD8^+^ DEG) and Supplemental Data 3 (CD4^+^ DEG).

### ATAC-seq

For each sample, 1000–20,000 cells were sorted into FACS buffer and Tn5 transposition was performed^79^. Briefly, cells were resuspended in 12.5 μl 2× tagmentation DNA Buffer, 2.5 μl Tn5, 2.5 μl 1% Tween-20, 2.5 μl 0.2% Digitonin, and 5 μl H_2_O and incubated at 37 C for 1 h. Cells were then lysed with the addition of 2 μl 10 mg/ml Proteinase-K, 23 μl Tagmentation Clean-up buffer (326 mM NaCl, 109 mM EDTA, 0.63% SDS), and incubated at 40 °C for 30 min. Tagmented DNA was purified and size selected for small fragments using AMPure XP beads (Beckman Coulter, A63881) and PCR amplified (Roche, KK2602) with dual indexing primers (Illumina, FC-131- 2004) to generate a sequencing library. Final libraries were again purified, and size selected using AMPureXP beads, quantitated by QuBit (Life Technologies, Q33231), size distributions determined by bioanalyzer (Agilent 2100), pooled at equimolar ratios, and sequenced at the Emory Non-human Primate Genomics Core on a NovaSeq6000 using a PE100 run.

### ATAC-seq data analysis

Raw sequencing data was mapped to the hg38 genome using Bowtie v1.1.1^80^. Peaks of accessibility enrichment were called using MACS2 v2.1.0^81^ and annotated to the nearest gene using HOMER^43^. Differential testing of accessible peak regions was performed using DESeq2 using the cutoffs of FDR ≤ 0.05 and >1.5 log_2_ fold-change to establish significance. Differentially accessible regions are listed in Supplemental Data 4. Count-based motif enrichment analysis was performed using the chromVAR^31^ R package. For this analysis, peaks identified by MACS2 were restricted to a fixed width of 250 bp using the ‘resize’ function from the GenomicRanges^74^ package in R. A matrix of counts for fragment insertions within these peaks was then generated using the ‘getCounts’ function from the ChromVAR package using the previously mapped sequencing reads. Overlapping peaks or those containing no fragment counts were excluded from the analysis and the motifs for transcription factor binding sites were sourced from the JASPAR^82^ database. Discovery of patterned accessibility regions (PAR) was performed using a custom R script, which used FDR cutoffs in either resting or stimulated comparisons. For example, primed-PAR were defined as significantly differential between stimulated and unstimulated naïve cells with a non-zero log_2_FC as well as significant in differential comparison between unstimulated naïve and MTC. A list of all PAR assignments for each locus can be found in Supplemental Data 5. The HOMER ‘findMotifsGenome.pl’ function was used for de novo motif enrichment analysis or known motif enrichment from this database. Relative enrichment rank change values were calculated using HOMER by normalizing the enrichment p-values for individual motifs in each set of peaks by the total number of enriched motifs found in that peak set. Resulting values were than z-score scaled for relative comparison across groups.

### Integrated analysis and statistics

For principal component analysis, normalized count data was mean scaled by row (across samples) and then analyzed using the ‘princomp’ function from the stats package in R. Phylogenetic analysis was performed by computing a Euclidean distance matrix between enrichment rank values and clustering using the ‘hclust’ function in R. Trees were plotted with APE v3.4^83^ as unrooted trees. Bootstrapping was used to assess the reproducibility of clustering using the ‘boot.phylo’ function with 10,000 permutations. PageRank^84^ analysis was performed using both normalized differential expression values derived from the RNA-seq data as well as raw ATAC-seq data. All statistical analyses were performed with R using DESeq2 for large-scale statistical analysis of RNA-seq or ATAC-seq data or using Wilcoxon Rank sum tests on individual genes with *p* ≤ 0.05 considered significant.

For all metabolic assays, a one-way ANOVA with multiple comparisons was used to determine significance. Three to six independent samples were used in these assays as indicated in the legends. Values *p* ≤ 0.05 were considered significant.

### Metabolic flux analysis (Seahorse assay)

ECAR was measured with XF96 Extracellular Flux Analyzer (Seahorse Bioscience). Human PBMCs isolated from three different donors. T cells were enriched by using human Pan T-cell isolation kit (Miltenyi Biotec). Cells were stained with antibodies to CD3 (BV450), CD4 (APC), CD8 (APC-Cy7), CD45RA (BV650), CCR7 (AF488) and ghost viability dye (BV510). Then, CD4^+^ (naïve, T_EM_, T_CM_) populations were sorted by FACS. The cells were cultured in RPMI media supplemented with IL-7 and IL-15 for 2 days with or without anti-CD3/CD28 beads (1:2 ratio). Cells were harvested, washed, and then resuspended in XF RPMI media. Cells (180,000–250,000) were transferred into a poly-D-lysine coated 96-well plate as three technical repeats for each cell type and centrifuged at 400xg for 5 min to allow the cells to collect into monolayer at the bottom of the plate. The plate was incubated at 37 °C non-CO_2_ incubator for 1 h and then placed into the XF96 Extracellular Flux Analyzer. Cells were monitored under basal conditions and in response to 10 mM glucose, 1 μM oligomycin, 100 mM 2-Deoxy-Glucose. ECAR values were extracted from Agilent Seahorse Wave Desktop software and normalized according to the initial cell number. Glycolysis, glycolytic capacity and glycolytic reserve values were calculated according to the equations provided at Agilent Report Generator User Guide.

### Mitochondrial measurements

T cells were enriched from human PBMCs by using a human Pan T-cell isolation kit (Miltenyi Biotec). The cells were cultured in RPMI media supplemented with IL-7 and IL-15 for 2 days with or without anti-CD3/CD28 beads (1:2 ratio). For assessment of mitochondrial mass, the cells were incubated with 25 nM MitoTracker Green FM (MTG, Invitrogen) for 1 h. To investigate the mitochondrial membrane potential of the cells, TMRM dye (Invitrogen) was added to a final concentration of 100 nM for the last 30 min of the above MTG incubation. Cells were stained with anti- CD3 (BV450), CD4 (PE-Cy7), CD8 (APC-Cy7), CCR7 (APC), and CD45RA (AF700) antibodies. Unfixed samples were immediately analyzed, and all samples were analyzed on a BD Fortessa flow cytometer.

### SCENITH assays

T cells were enriched from freshly isolated human PBMCs. The cells were plated in a 96-well round bottom plate and rested at 37 °C, 5% CO_2_ for 2 h. Some samples were stimulated with anti-CD3/CD28 beads (2:1 ratio) for 1 day. If the cells were not stimulated, the SCENITH protocol was performed after the 2 h resting step. In the SCENITH protocol, cells were untreated or treated with 2-deoxy-glucose (250 mM, Sigma–Aldrich), oligomycin (1.5 µM, Sigma–Aldrich), or a combination of both drugs for 20 min at 37 °C. Puromycin (10 µg/ml, Sigma-Aldrich) was added, and the cells were incubated at 37 °C for another 25 min. The cells were immediately washed with FACS buffer, then the surface staining was performed. The cells were incubated with anti-CD3 (V450), CD4 (PE-Cy7), CD8 (APC-Cy7), CCR7 (FITC), CD45RA (AF700) antibodies and ghost viability dye (BV510) in FACS buffer at 4 °C for 20 min. After washing with FACS buffer, the cells were fixed and permeabilized using Foxp3 intracellular staining kit (Invitrogen eBioscience) for 1 h at room temperature. Intracellular staining of puromycin was performed by using 1:1100 dilution of anti-puromycin (AF647) antibodies (Clone 12D10, Sigma-Aldrich) in kit supplied perm/wash buffer. The cells were incubated at room temperature for one hr, and then washed with perm/wash buffer twice. Finally, the cells were resuspended in FACS buffer and analyzed by flow cytometry. The mitochondrial dependence, glucose dependence, glycolytic capacity, fatty acid, and amino acid oxidation capacity (FAO and AAO) were calculated from the MFI of puromycin in the different treatments^28^ with adjustment of range values across samples within a group if a value within the group was negative. Briefly, percent mitochondrial dependence is calculated as the difference between control and oligomycin-treated cells divided by the difference between control and 2-deoxy-glucose plus oligomycin-treated cells. Percent glucose dependence was calculated as difference between control and 2-deoxy-glucose-treated cells divided by the difference between control and 2-deoxy-glucose plus oligomycin-treated cells. Glycolytic capacity and FAO and AAO capacity were calculated by subtracting the percent mitochondrial capacity or percent glycolytic capacity from 100, respectively.

### Detection of intracellular proteins by flow cytometry

For intracellular cytokine detection experiments, T cells were enriched from PBMC as above and stimulated for 48 h with anti-CD3/CD28 beads (2:1 ratio). The cells were incubated with both Golgi Stop (BD Biosceinces) and Brefaldin A (Biolegend) for 5 h at 37 °C. Surface staining was performed with washed cells using by anti- CD3 (BV605), CD4 (BV785), CD8 (APC-Cy7), CCR7 (AF488), CD45RA (AF700) antibodies and ghost viability dye (BV510). After washing with FACS buffer, cells were fixed and permeabilized for 20 min using the Cytofix/Cytoperm kit (BD Bioscience). The cells were again washed with permeabilization buffer. Intracellular staining was performed to detect the cytokines by using IL-1α (PE), IL-4 (APC), IL-2 (PE-Cy7), IL-5 (eflour450), IFNγ (BV711), TNFα (BV650), IL-22 (BUV737), and IL-17 (PerCP) antibodies in permeabilization buffer. The isotype controls used were Mouse IgG1, κ Isotype (PE), Rat IgG1, κ Isotype (APC), Rat IgG2a, κ Isotype (PE/Cy7), Rat IgG1 κ Isotype (eflour450), Mouse IgG1, κ Isotype (BV711), Mouse IgG1, κ Isotype (BV650), Mouse IgG1 κ Isotype (BUV737), Mouse IgG1 (PerCP). The cells were incubated at 4 °C for 30 min, and then washed with permeabilization buffer and resuspended in FACS buffer. The samples were analyzed by flow cytometry.

To detect the transcription factors levels in unstimulated and stimulated T cells (with anti-CD3/CD28 beads for 2 days), surface staining was performed with anti-CD3 (BV650), CD4 (BV711), CD8 (APC-Cy7), CCR7 (AF488), and CD45RA (AF700) for 20 min at 4 °C. After washing the cells, they were fixed and permeabilized using the Foxp3 intracellular staining kit as above. Intracellular staining was done using antibodies to EOMES (PE-Cy5.5), T-BET (BV421), HIF1α (APC), and AHR (PE-Cy7). Following staining for MSC, cells were restained with an anti-rabbit secondary antibody (PE). The isotype controls used were Mouse IgG1 κ Isotype (PE/Cy5.5), Mouse IgG1, κ Isotype (BV421), Mouse IgG1 (APC), Mouse IgG2b κ Isotype (PE/Cy7), Rabbit IgG. The cells were washed with permeabilization buffer and resuspended in FACS buffer for flow cytometry analysis.

### Reporting summary

Further information on research design is available in the Nature Portfolio Reporting Summary linked to this article.

## Supplementary information

Supplementary Information

Description of Additional Supplementary Files

Supplemental Data 1

Supplemental Data 2

Supplemental Data 3

Supplemental Data 4

Supplemental Data 5

Supplemental Data 6

Reporting Summary

## Data Availability

All sequencing data have been deposited in NCBI Gene Expression Omnibus (GEO) under the following accession numbers GSE186463 for RNA-seq and GSE186462 for ATAC-seq. Raw data used to create Fig. 1f, 2b, l, 3c, d, f, h-p, 5i, 7g, h, 8d–g, 9b, e, f, and h are included in Supplemental Data 6.

## Code availability

Code and data processing scripts are available from the corresponding author upon request and at https://github.com/cdschar/Rose_MTC_genomics or at 10.5281/zenodo.7692608.
