# Supplementary material for: Distinct transcriptomic and epigenomic modalities underpin human memory T cell subsets and their activation potential
Source: Commun Biol. 2023 Apr 3;6:363. doi: 10.1038/s42003-023-04747-9 (PMC10070634; doi:10.1038/s42003-023-04747-9)
Supplement: Supplementary file 1 — Supplementary Information [file 42003_2023_4747_MOESM1_ESM.pdf]

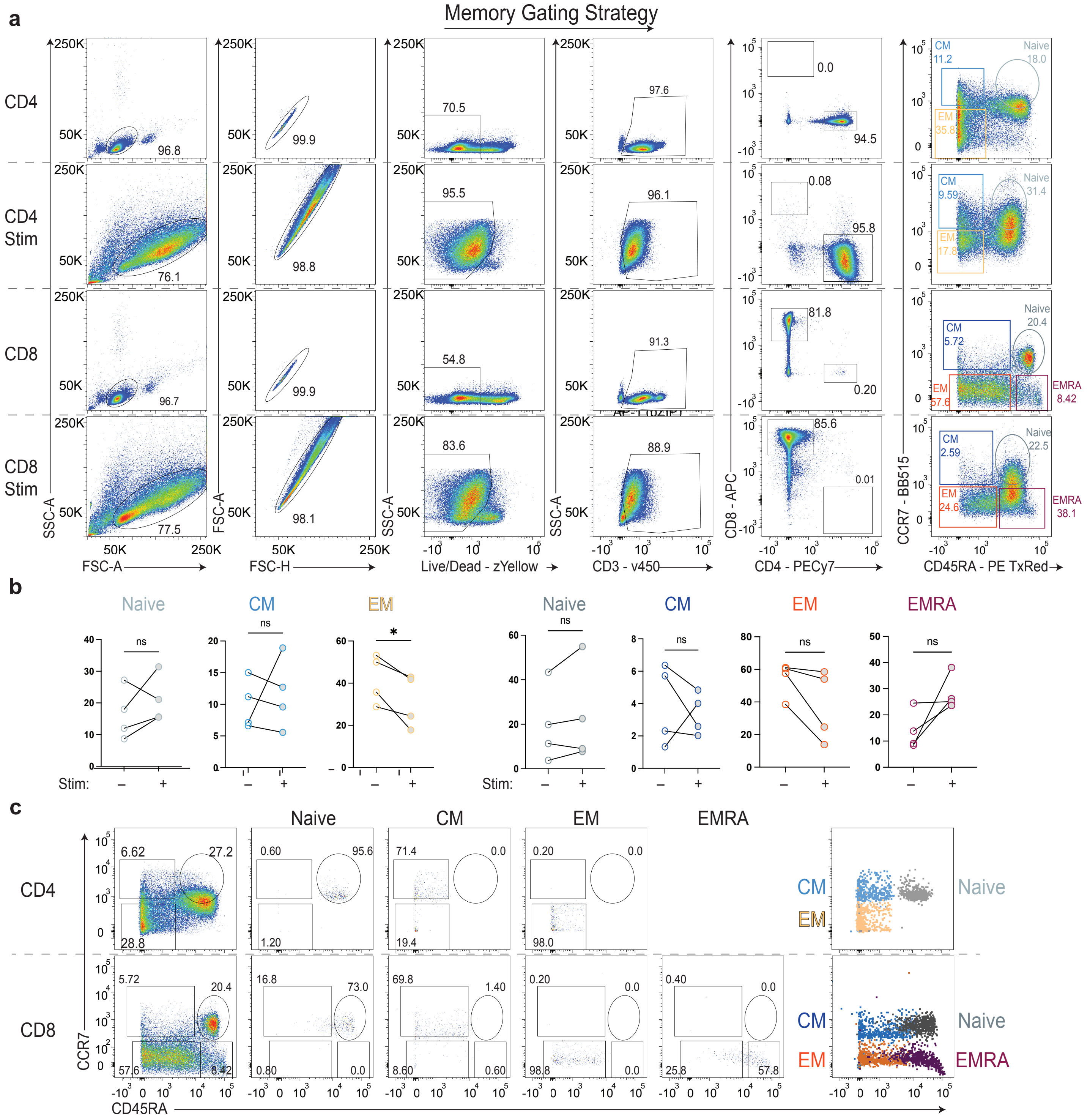

Supplemental Figure 1. **(a)** Representative flow cytometry plots showing gating strategy used to sort CD4+ (top two rows) and CD8+ (bottom two rows) memory cells T cells. **(b)** Frequency of MTC subsets in resting and stimulated samples for CD4+ (left) and CD8+ (right) T cells (*p < 0.05, paired t-test). **(c)** Post-sort analysis, including overlaid CCR7 by CD45RA scatter (last column), of MTC subsets depicting purity of sorted populations.

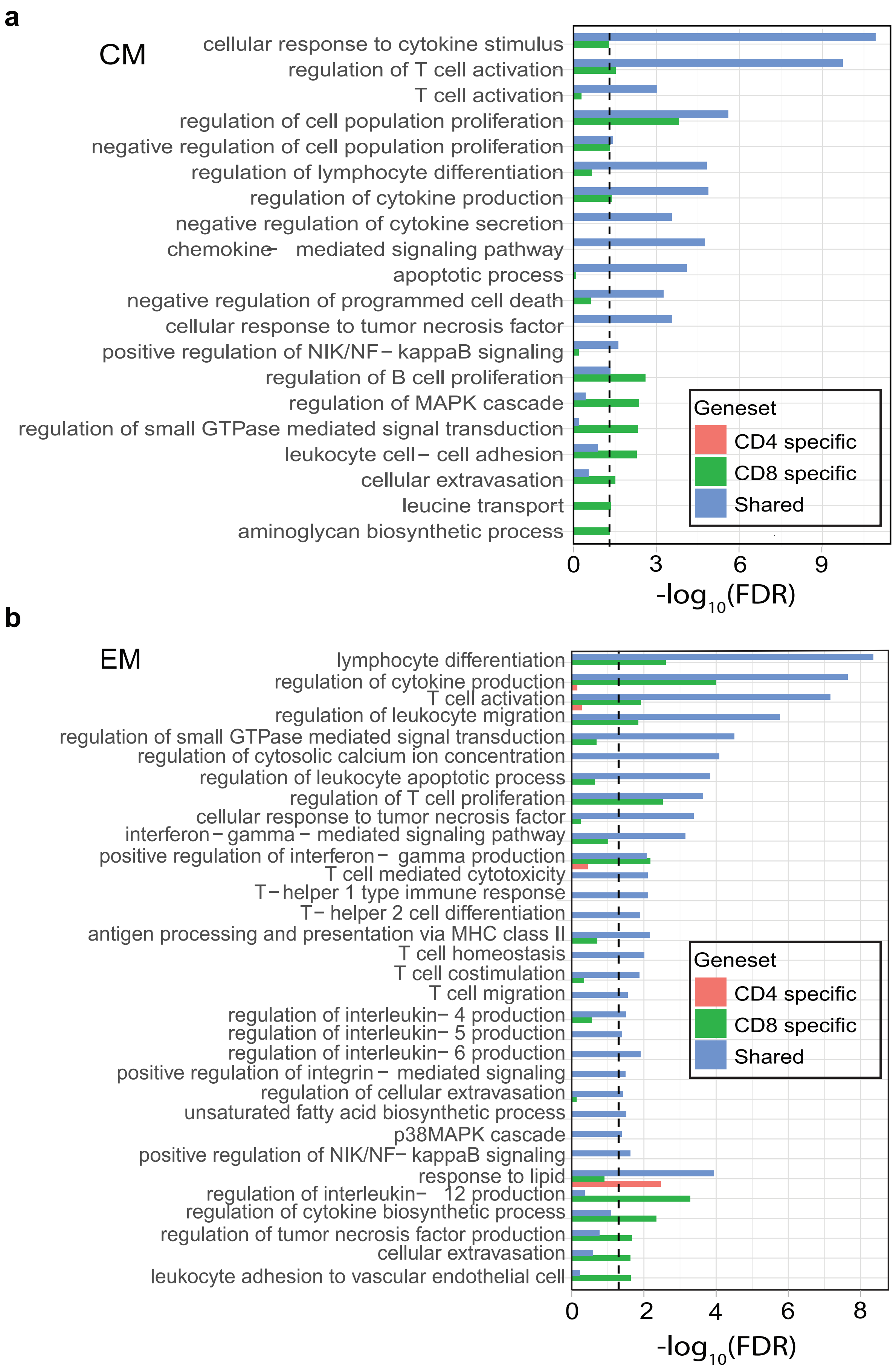

Supplemental Figure 2. (a) Bar plot showing FDR-corrected p-value of enriched gene ontology pathways representing CD4+ specific, CD8+ specific and shared DEGs for TCM. Significant enrichment level marked with dotted line. **(b)** Same as A for TEM.

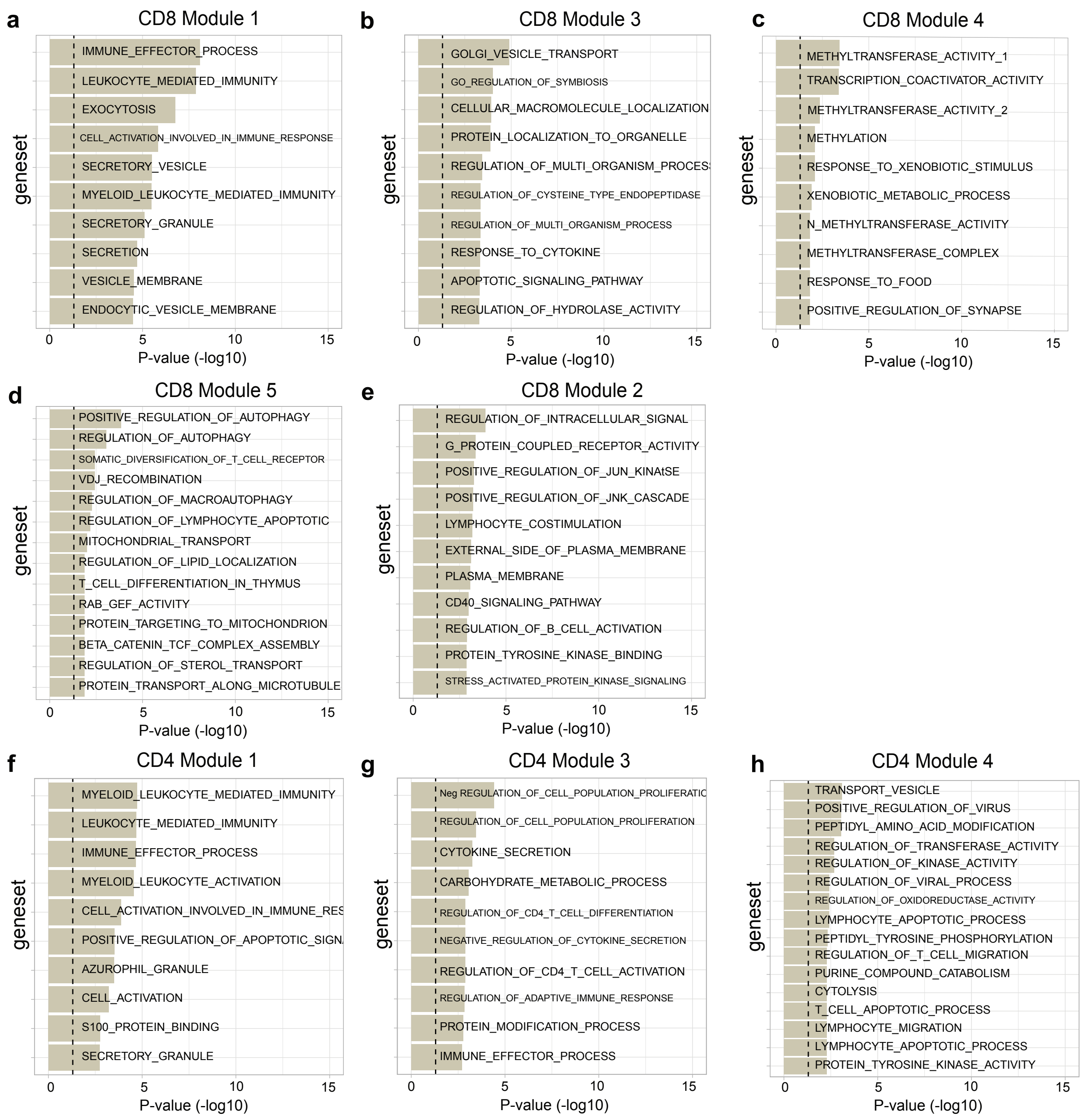

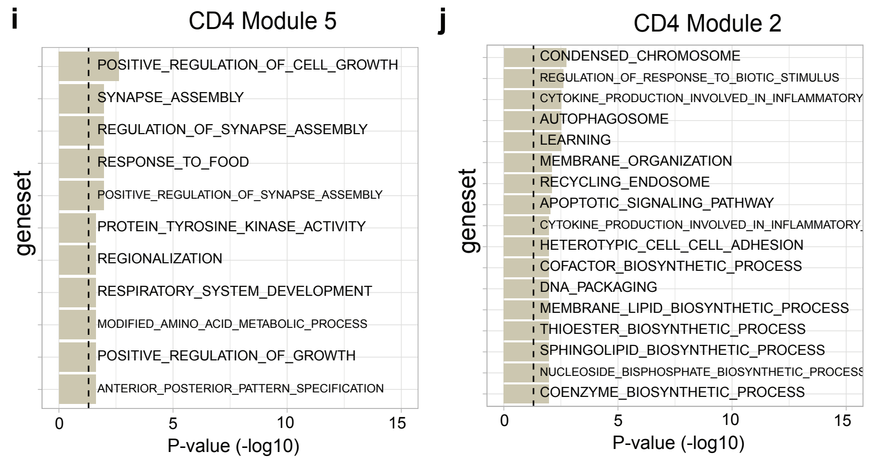

Supplemental Figure 3. (a-e) Bar plots showing FDR-corrected p-value of enriched gene ontology pathways for individual gene modules as labeled in CD8+ T cells. Significant enrichment level marked with dotted line. **(f-j)** CD4+ T cells.

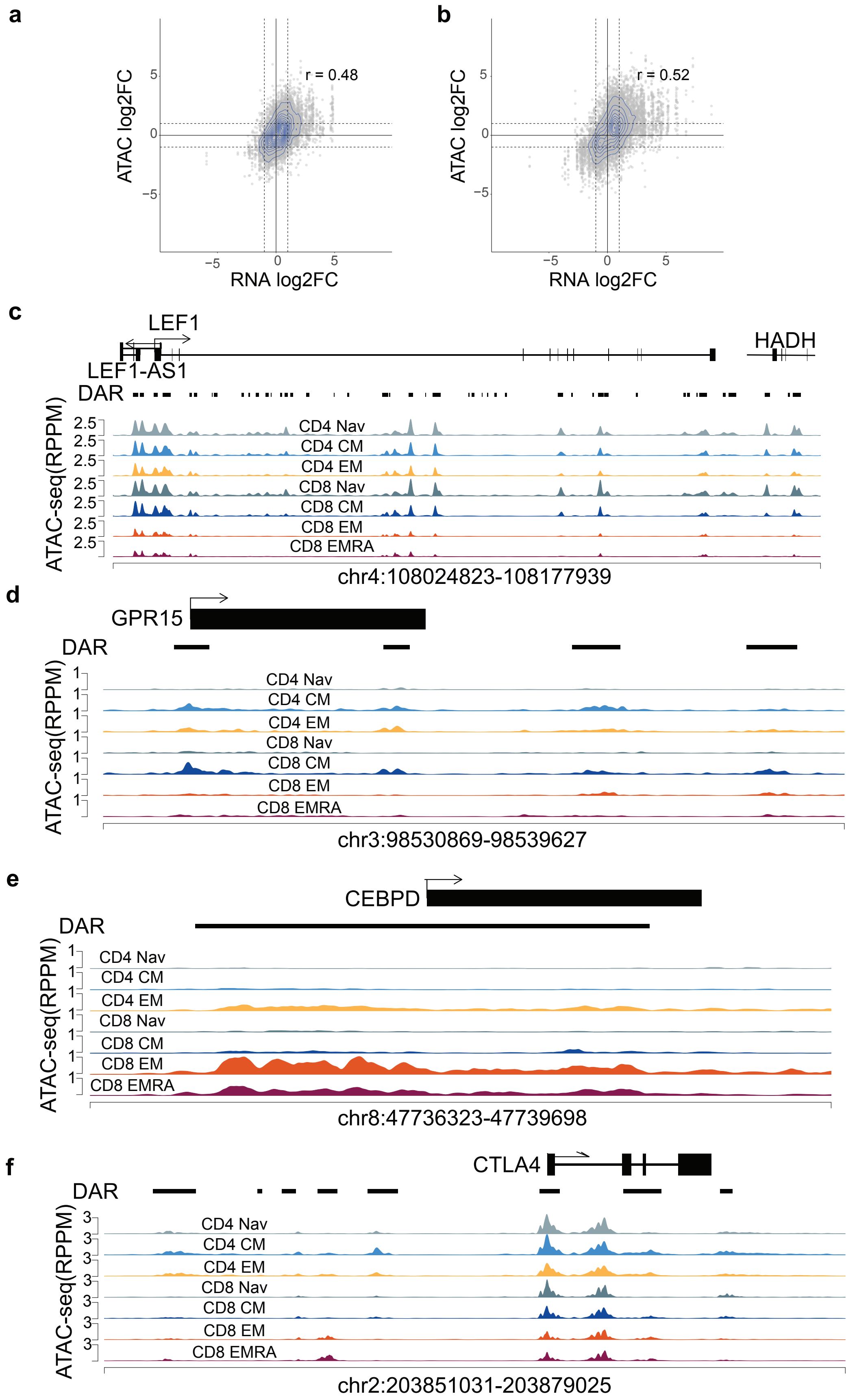

Supplemental Figure 4. (a) Scatter plot showing intersection of DEG and DAR for CD4+ TCM. Dotted lines represent ±1 log2FC. **(b)** Same as (a) for CD4+ TEM. **(c-f)** Genome plots showing the average accessibility levels in each of the memory subsets and naïve T cells at the indicated locus. Data represent the mean of each cell type. DAR are highlighted by black boxes at the top of plots.

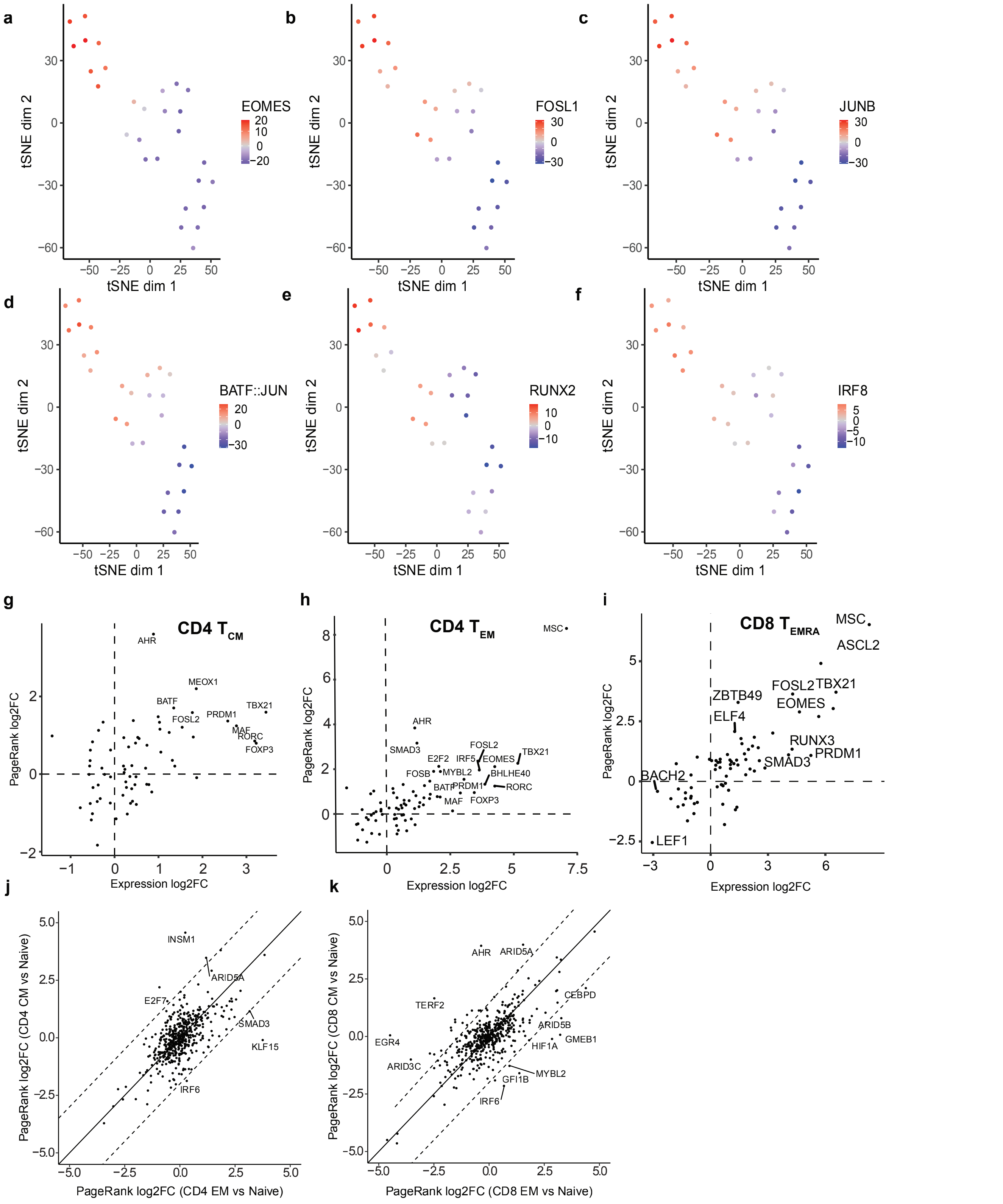

Supplemental Figure 5. **(a-f)** tSNE plot from Figure 5b colored by variability around indicated binding motifs. **(g-i)** Scatter plots showing log2 fold changes (log2FC) of RNA expression (x-axis) and PageRank statistic log2FC (y-axis) between memory and naïve samples for labeled CD8+ memory subsets. **(j-k)** Scatter plots of log2FC PageRank statistic (vs Naïve) for TEM (x-axis) vs TCM (y-axis) in CD4+ **(j)** and CD8+ **(k)**. Solid line represents unity line. Dotted lines represent 1 log2FC away from unity.

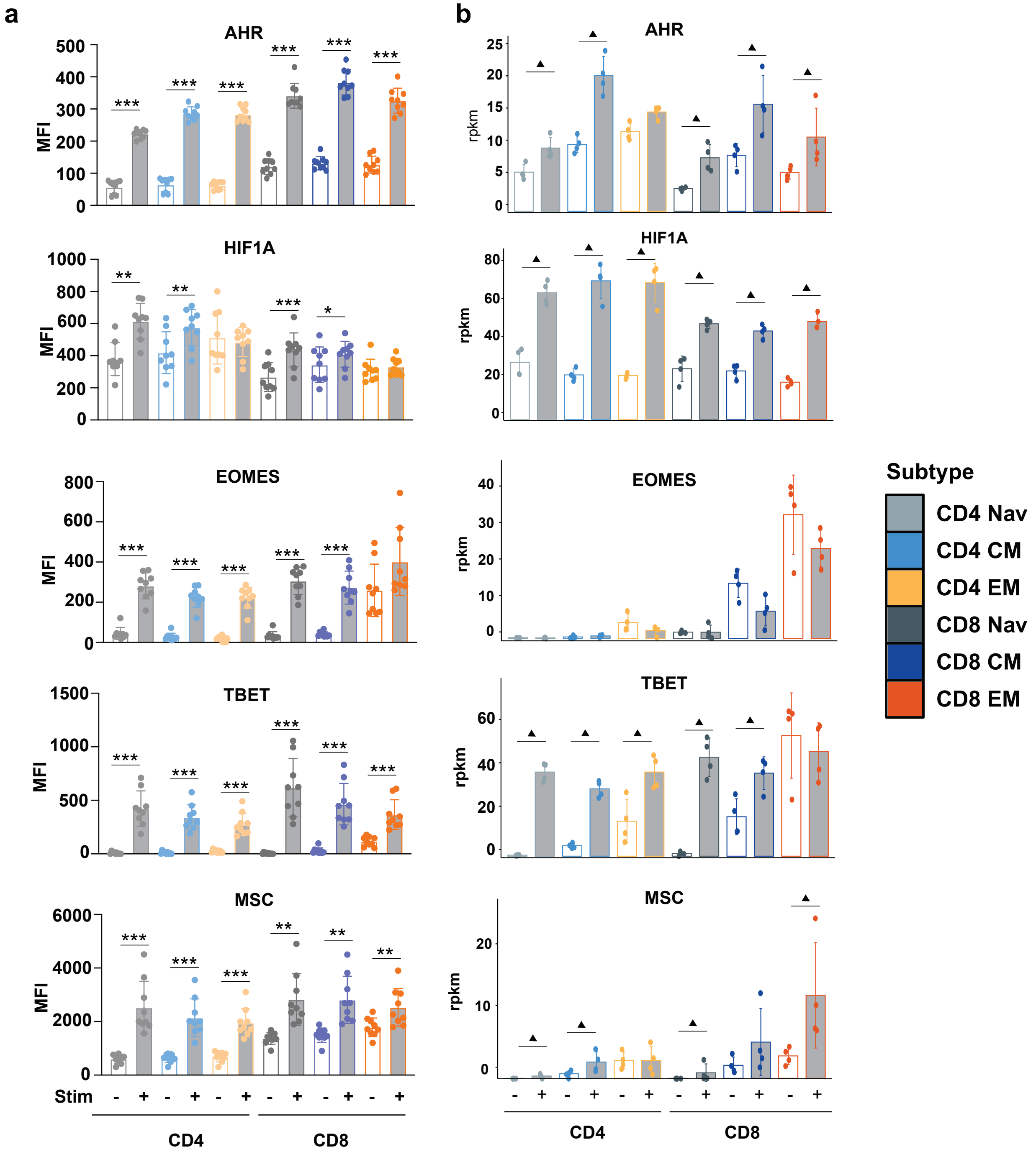

Supplemental Figure 6. (a) Median fluorescence intensity (MFI) of corresponding transcription factors in unstimulated and stimulated MTC measured by intracellular staining and flow cytometry. Isotype controls substracted as background. N=9, *P<0.05, **P<0.01, ***P<0.001. Paired t-test was performed. **(b)** RNA expression data of the transcription factors for MTC subsets unstimulated and stimulated expressed as reads per kilobase million (rpkm). Statistical analyses were performed using DEseq2. N=4, ▲, FDR < 0.05 and log2FC > 1.

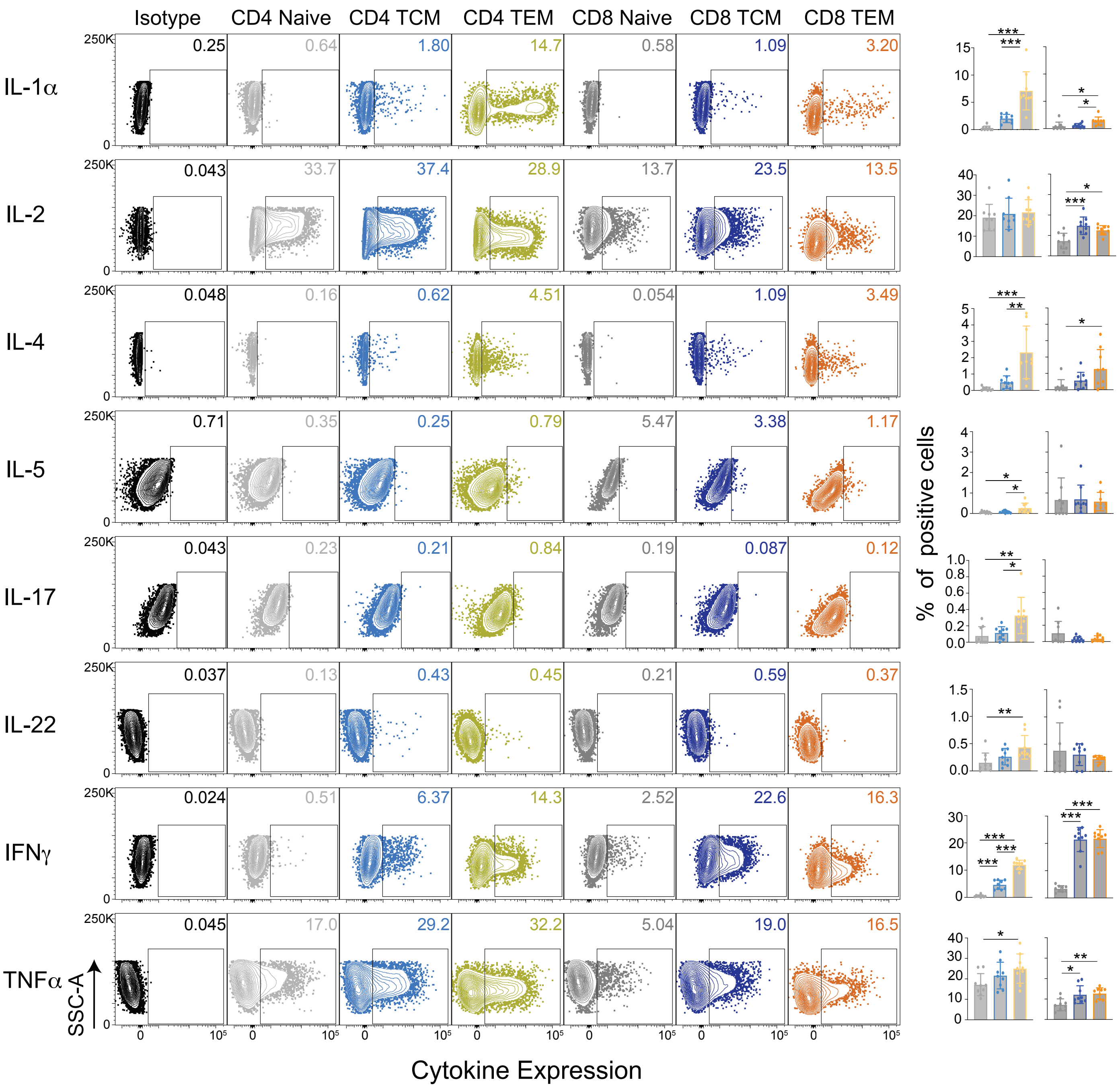

Supplemental Figure 7. Cytokines expression was measured in stimulated T cells by intracellular staining and flow cytometry. Left, representative flow cytometry plots showing data and gating strategy. Right, bar plots showing the frequency of the cells that express the corresponding cytokine. N=9, *P<0.05, **P<0.01, ***P<0.001. One way ANOVA, multiple comparisons was performed to determine significant differences.

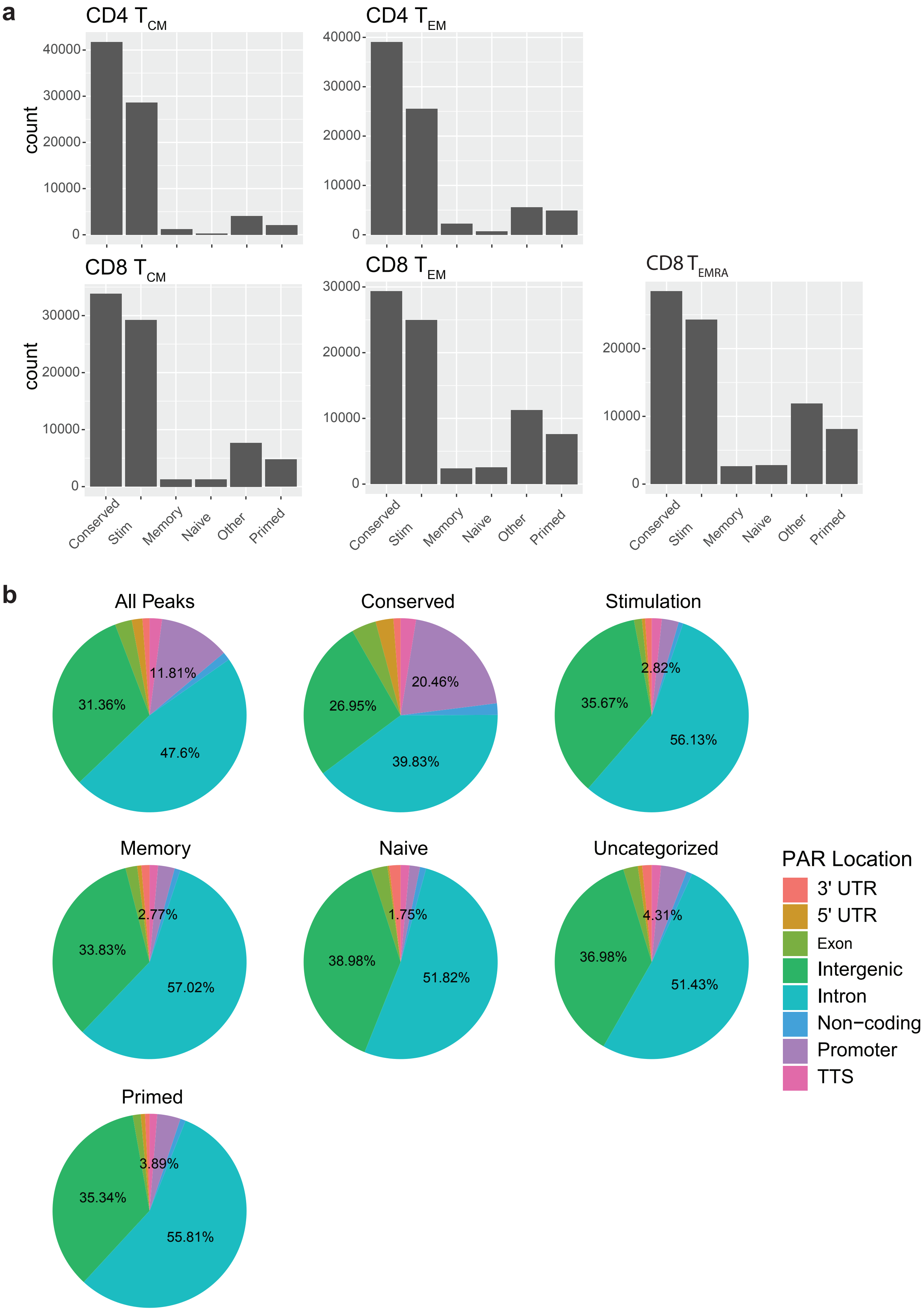

Supplemental Figure 8. (a) Histograms of counts for PAR categories for indicated MTC subsets. **(b)** Pie charts for indicated PAR categories showing percentages of genomic annotations associated with each.

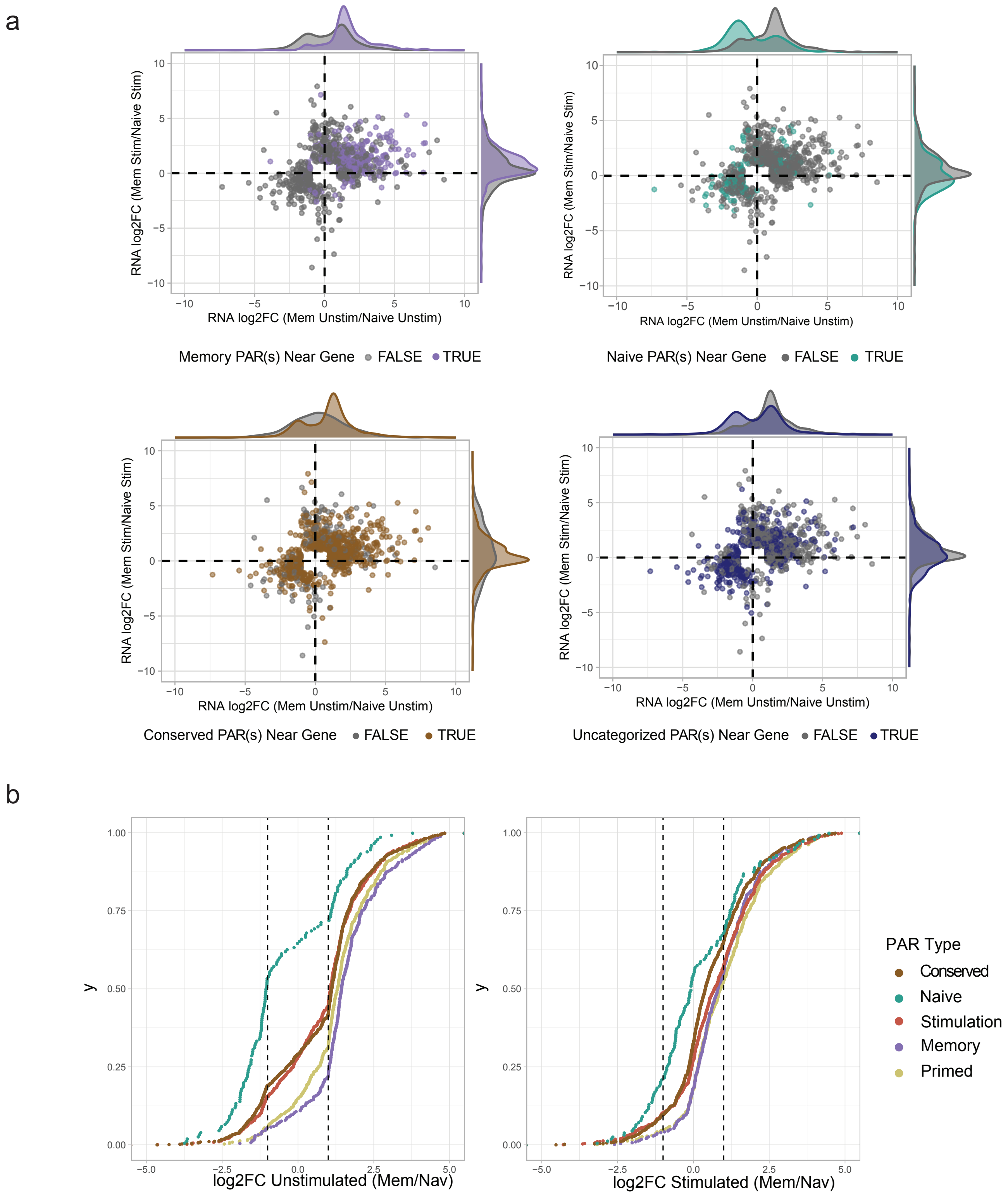

Supplemental Figure 9. **(a)** Scatter plot showing log2-transformed fold changes (log2FC) for DEG found to be significant in either unstimulated memory vs unstimulated naïve T cells (x-axis) or stimulated memory vs stimulated naïve T cells (y-axis). Dots are colored in respective colors if there is at least one PAR mapped to differential gene locus. **(b)** Empirical cumulative distribution function plots for unstimulated DEG (left) and stimulated DEG (right) colored by presence of different PAR categories.

Supplementary Table 1: List of Antibodies				
**Antibody Name**	**Fluorophore**	**Catalog Number**	**Company**	**Used Concentration (µg/mL)**
CD3	BV605	300459	Biolegend	1.3
CD3	BV650	317324	Biolegend	0.2
CD3	BV450	75-0037-T100	Tonbo	1.3
CD4	BV711	344648	Biolegend	1.3
CD4	BV785	317441	Biolegend	1
CD4	PE/Cyanine7	344612	Biolegend	1.3
CD4	PE/Cyanine7	300511	Biolegend	10
CD8	APC/Cyanine7	344714	Biolegend	1.3
CD8	AF488	MHCD0801	Thermofisher	0.2
CCR7	APC	353213	Biolegend	2.6
CCR7	AF488	353206	Biolegend	5
CCR7	BB515	565870	BD Biosciences	10
CD45RA	AF700	560673	BD Biosciences	1.3
CD45RA	BV650	740608	BD Biosciences	2.6
CD45RA	PE/TxRed	304145	Biolegend	2
MSC Polyclonal		PA5-118504	Thermofisher	10
Rabbit Polyclonal Isotype Ctrl		910801	Biolegend	10
Donkey anti-rabbit IgG	PE	406421	Biolegend	0.87
AHR	PE/Cyanine7	25-9854-42	Thermofisher	2
Mouse IgG2b, κ Isotype Ctrl	PE/Cyanine7	25-4732-81	Thermofisher	2
HIF-1 alpha	APC	IC1935A	R&D Sysytems	0.2
Mouse IgG1 Isotype Ctrl	APC	IC002A	R&D Sysytems	0.2
T-bet	BV421	644815	Biolegend	1.2
Mouse IgG1, κ Isotype Ctrl	BV21	400157	Biolegend	1.2
EOMES	PE-Cyanine5.5	35-4877-42	Thermofisher	1
Mouse IgG1, κ Isotype Ctrl	PE-Cyanine5.5	35-4714-82	Thermofisher	1
Puromycin	AF647	MABE343-AF647	Millipore Sigma	0.45
IL-1α	PE	500106	Biolegend	0.8
Mouse IgG1, κ Isotype Ctrl	PE	400111	Biolegend	0.8
IL-4	APC	500812	Biolegend	0.25
Rat IgG1, κ Isotype Ctrl	APC	400411	Biolegend	0.25
IL-2	PE/Cyanine7	500326	Biolegend	0.5
Rat IgG2a, κ Isotype Ctrl	PE/Cyanine7	400521	Biolegend	0.5
IL-5	eFluour 450	48-7052-82	Thermofisher	4
Rat IgG1, κ Isotype Ctrl	eFluour 450	48-4301-80	Thermofisher	4
IFN-γ	BV711	502539	Biolegend	1
Mouse IgG1, κ Isotype Ctrl	BV711	400167	Biolegend	1
TNF-α	BV650	502937	Biolegend	2
Mouse IgG1, κ Isotype Ctrl	BV650	400163	Biolegend	2
IL-22	BUV737	367-7229-42	Thermofisher	1
Mouse IgG1, κ Isotype Ctrl	BUV737	367-4714-81	Thermofisher	1
IL-17/IL-17A	PerCP	IC3171C-025	R&D systems	1
Mouse IgG1, Isotype Ctrl	PerCP	IC002C	R&D systems	1
