# Supplementary material for: Distinct transcriptomic and epigenomic modalities underpin human memory T cell subsets and their activation potential
Source: Commun Biol. 2023 Apr 3;6:363. doi: 10.1038/s42003-023-04747-9 (PMC10070634; doi:10.1038/s42003-023-04747-9)
Supplement: Supplementary file 2 — Description of Additional Supplementary Files [file 42003_2023_4747_MOESM2_ESM.pdf]

Description of Additional Supplementary Files

File name: Supplemental Data 1
Description: Differentially expressed Genes.

File name: Supplemental Data 2
Description: Clustering modules for CD8 T cells.

File name: Supplemental Data 3
Description: Clustering modules for CD4 T cells.

File name: Supplemental Data 4
Description: Differentially Accessible Regions.

File name: Supplemental Data 5
Description: Patterned accessibility regions classifications.
