# Supplementary material for: Distinct transcriptomic and epigenomic modalities underpin human memory T cell subsets and their activation potential
Source: Commun Biol. 2023 Apr 3;6:363. doi: 10.1038/s42003-023-04747-9 (PMC10070634; doi:10.1038/s42003-023-04747-9)
Supplement: Supplementary file 9 — Reporting Summary [file 42003_2023_4747_MOESM9_ESM.pdf]

nature portfolio

Corresponding author(s): Jeremy M. Boss

Last updated by author(s): Feb 1, 2023

Reporting Summary

Nature Portfolio wishes to improve the reproducibility of the work that we publish. This form provides structure for consistency and transparency in reporting. For further information on Nature Portfolio policies, see our Editorial Policies and the Editorial Policy Checklist.

Statistics

For all statistical analyses, confirm that the following items are present in the figure legend, table legend, main text, or Methods section.

n/a	Confirmed	
☐	☒	The exact sample size (*n*) for each experimental group/condition, given as a discrete number and unit of measurement
☐	☒	A statement on whether measurements were taken from distinct samples or whether the same sample was measured repeatedly
☐	☒	The statistical test(s) used AND whether they are one- or two-sided<br>*Only common tests should be described solely by name; describe more complex techniques in the Methods section.*
☐	☒	A description of all covariates tested
☐	☒	A description of any assumptions or corrections, such as tests of normality and adjustment for multiple comparisons
☐	☒	A full description of the statistical parameters including central tendency (e.g. means) or other basic estimates (e.g. regression coefficient) AND variation (e.g. standard deviation) or associated estimates of uncertainty (e.g. confidence intervals)
☐	☒	For null hypothesis testing, the test statistic (e.g. F, t, r) with confidence intervals, effect sizes, degrees of freedom and P value noted<br>*Give P values as exact values whenever suitable.*
☒	☐	For Bayesian analysis, information on the choice of priors and Markov chain Monte Carlo settings
☒	☐	For hierarchical and complex designs, identification of the appropriate level for tests and full reporting of outcomes
☐	☒	Estimates of effect sizes (e.g. Cohen's *d*, Pearson's *r*), indicating how they were calculated

Our web collection on statistics for biologists contains articles on many of the points above.

Software and code

Policy information about availability of computer code

Data collection	Flow cytometry data was collected using BD FACSDiva software (BD Biosciences)
Data analysis	For flow cyometry, data were analyzed using FlowJo v 10.6.2. Raw sequencing data were mapped using STAR v2.5.3, and analyzed for duplicates using PICARD. The following programs were used to pre-process or analyze the data: GSEA v4.0, MACS2 v2.1.0, Bowtie v1.1.1, APE v3.4, and HOMER v4.11. Custom code was used for further analysis utilizing the following R programing packages: GenmicRanges, DESeq2, princomp, hclust, e1071, and chromVAR. Custom code was used for data visualization utilizing the following R programming packages: ggplot2, ComplexHeatmap, Triwise. All custom code used can be found at https://github.com/cdschar/Rose_MTC_genomics

For manuscripts utilizing custom algorithms or software that are central to the research but not yet described in published literature, software must be made available to editors and reviewers. We strongly encourage code deposition in a community repository (e.g. GitHub). See the Nature Portfolio guidelines for submitting code & software for further information.

Data

Policy information about availability of data

All manuscripts must include a data availability statement. This statement should provide the following information, where applicable:

- Accession codes, unique identifiers, or web links for publicly available datasets
- A description of any restrictions on data availability
- For clinical datasets or third party data, please ensure that the statement adheres to our policy

All sequencing data have been deposited in NCBI Gene Expression Omnibus (GEO) under the following accession numbers GSE186463 for RNA-seq and GSE186462 for ATAC-seq.

Human research participants

Policy information about studies involving human research participants and Sex and Gender in Research.

Reporting on sex and gender	Patient samples in this study were de-identified as the subject of this work was differences between cell types and not individuals
Population characteristics	No characteristic information for the recruited individuals was collected as donors were de-identified according to protocol.
Recruitment	Healthy volunteer donors were recruited via Emory University School of Medicine in a de-identified manner.
Ethics oversight	Emory University School of Medicine Institutional Review Board protocols, IRB00045821

Note that full information on the approval of the study protocol must also be provided in the manuscript.

Field-specific reporting

Please select the one below that is the best fit for your research. If you are not sure, read the appropriate sections before making your selection.

☒ Life sciences ☐ Behavioural & social sciences ☐ Ecological, evolutionary & environmental sciences

For a reference copy of the document with all sections, see nature.com/documents/nr-reporting-summary-flat.pdf

Life sciences study design

All studies must disclose on these points even when the disclosure is negative.

Sample size	Four replicates was chosen for each isolated cell type in sequencing assays to establish the variation around individuals. For metabolic assays 3-6 individual replicates were chosen to provide sufficient power.
Data exclusions	Sequencing data collected for the CD4+ EMRA cell type was excluded after quality control analysis indicated that isolation flow gating was not sufficient to distinguish this population from the Naive CD4+ T cells.
Replication	Several results were replicated from previous work, or between sequencing assays. Metabolic analysis were replicated twice.
Randomization	In this study distinct cell types serve as the experimental group so randomization was not necessary
Blinding	Data collection was done blind of human donors. As the relevant comparisons are all within individuals in this study no covariates were needed across individuals.

Reporting for specific materials, systems and methods

We require information from authors about some types of materials, experimental systems and methods used in many studies. Here, indicate whether each material, system or method listed is relevant to your study. If you are not sure if a list item applies to your research, read the appropriate section before selecting a response.

Materials & experimental systems

n/a	Involved in the study	
☐	☒	Antibodies
☒	☐	Eukaryotic cell lines
☒	☐	Palaeontology and archaeology
☒	☐	Animals and other organisms
☒	☐	Clinical data
☒	☐	Dual use research of concern

Methods

n/a	Involved in the study	
☒	☐	ChIP-seq
☐	☒	Flow cytometry
☒	☐	MRI-based neuroimaging

Antibodies

Antibodies used

Antibodies used for metabolic and intracelluar protein analysis were: CD3-BV450 (Tonbo Biosciences: 75-0038), CD3-BV605 (Biolegend: 300459), CD3-BV650 (Biolegend: 317324), CD4-BV711 (Biolegend: 344648), CD4-BV785 (Biolegend: 317441), CD4-APC (Tonbo Biosciences:20-0048), CD4-PE/Cy7 (Biolegend: 344612), CD8-APC/Cy7 (Biolegend: 344714), CD45RA-BV650 (BD biosciences: 740608), CD45RA-AF700 (BD biosciences: 560673), CCR7-AF488 (Biolegend: 353206), CCR7-APC (Biolegend: 353213), puromycin-AF647 (Sigma-Aldrich: MABE343-AF647), IL1a-PE (Biolegend: 500106), Mouse IgG1, κ Isotype-PE (Biolegend: 400111), IL4-APC (Biolegend: 500812), Rat IgG1, κ Isotype-APC (Biolegend: 400411), IL2-PE/Cy7 (Biolegend: 500326), Rat IgG2a, κ Isotype-PE/Cy7 (Biolegend: 400521), IL5-eflour450 (Thermofisher: 48-7052-82), IgG1 κ Isotype-eflour450 (Thermofisher: 48-4301-80), IFNg-BV711 (Biolegend: 502539), Mouse IgG1, κ Isotype-BV711 (Biolegend: 400167) , TNFa-BV650 (Biolegend: 502937), Mouse IgG1, κ Isotype-BV650 (Biolegend: 400163), IL22-BUV737 (Thermofisher: 367-7229-42), Mouse IgG1 κ Isotype-BUV737 (Thermofisher: 367-4714-81), IL17-PerCP (R&D systems: IC3171C-025), Mouse IgG1-PerCP (R&D systems: IC002C), EOMES-PE/Cy5.5 (Thermofisher: 35-4877-42), Mouse IgG1 κ Isotype-PE/Cy5.5 (Thermofisher: 35-4714-82), TBET-BV421 (Biolegend: 644815), Mouse IgG1, κ Isotype-BV421 (Biolegend: 400157), HIF1a-APC (R&D systems: IC1935A), Mouse IgG1-APC (R&D systems: IC002A), AHR-PE/Cy7 (Thermofisher: 25-9854-42), Mouse IgG2b κ Isotype-PE/Cy7 (Thermofisher: 25-4732-81), MSC polyclonal antibody (Thermofisher: PA5-118504), Rabbit IgG-PE (Biolegend: 406421), Rabbit polyclonal isotype (Biolegend: 910801).

Validation

For all antibodies, manufacturers provide flow cytometry data of primary antibody vs isotype control for validation. In addition, we included isotype control antibodies for all intracellular stainings to eliminate any nonspecific interactions. All surface staining antibodies are commonly used ones and previously validated in our lab and in published studies.

Flow Cytometry

Plots

Confirm that:

☒ The axis labels state the marker and fluorochrome used (e.g. CD4-FITC).

☒ The axis scales are clearly visible. Include numbers along axes only for bottom left plot of group (a 'group' is an analysis of identical markers).

☒ All plots are contour plots with outliers or pseudocolor plots.

☒ A numerical value for number of cells or percentage (with statistics) is provided.

Methodology

Sample preparation

Whole blood samples were collected and enriched for PBMCs using density gradient centrifugations, treated with ACK lysing buffer to remove red blood cells, and washed in PBS. CD4+ and CD8+ T cells were enriched for using MACS microbead isolation kits via negative selection of non-targeted cells.

Instrument

Flow cytometry was performed on a FACSAria II (BD Biosciences)

Software

BD FACSDiva software (BD Biosciences) as well as FlowJO v10.6.2 were used to collect data. Data analysis was conducted using custom code which is available at https://github.com/cdschar/Rose_MTC_genomics

Cell population abundance

Post sort analysis indicated that relevant fractions accounted for 57-98% of the total sorted cells indicating good purity levels.

Gating strategy

Lymphocytes were gated based on SSC-A / FSC-A, single cells by FSC-H / FSC-A, and live cells were based on exclusion of Zombie Yellow Fixable Viability Kit. T cells of the appropriate linage were selected using the markers CD3, CD4, and CD8. Memory and naïve T cell subsets were isolated using the markers CCR7 and CD45RA.

☒ Tick this box to confirm that a figure exemplifying the gating strategy is provided in the Supplementary Information.
